# NAG-1/GDF15 as a tumor suppressor in colorectal cancer: inhibition of β-catenin and NF-κB pathways via interaction with EpCAM

**DOI:** 10.1038/s41419-025-07695-w

**Published:** 2025-05-02

**Authors:** Jaehak Lee, Ilju Kim, Junsun Ryu, Thomas Eling, Seung Joon Baek

**Affiliations:** 1 Laboratory of Signal Transduction, College of Veterinary Medicine and Research Institute for Veterinary Science, Seoul National University, Seoul, Korea; 2 Department of Otolaryngology-Head and Neck Surgery, Center for Thyroid Cancer, Research Institute and Hospital, National Cancer Center, Goyang-si, Gyeonggi-do Korea; 3 Retired Scientist Emeritus, NIEHS/NIH, Research Triangle Park, NC USA

**Keywords:** Growth factor signalling, Predictive markers

## Abstract

NAG-1/GDF15, a tumor suppressor, is synthesized as a pro-form in colorectal cancer (CRC) cells and undergoes cleavage to generate its mature form. While the biological function of pro-NAG-1/GDF15 remains unclear, our study reveals its crucial role in suppressing oncogenic signaling. We demonstrate that pro-NAG-1/GDF15 is predominantly retained within cells, whereas its mature form is secreted into the media. The expression of NAG-1/GDF15, or uncleavable R193A mutant, inhibits β-catenin and NF-κB signaling, key pathways in CRC progression. Mechanistically, the pro-NAG-1/GDF15 interacts with EpCAM, preventing its cleavage and nuclear translocation, thereby reducing β-catenin and NF-κB activity. This inhibition correlates with decreased expression of oncogenic targets such as cyclin D1 and c-myc. In vivo, NAG-1/GDF15 expression significantly reduces tumor growth in cancer xenograft models, accompanied by decreased proliferation and increased apoptosis. Furthermore, analysis of public datasets suggests that high NAG-1/GDF15 expression is associated with improved CRC patient survival. These findings highlight NAG-1/GDF15 via the formation of pro-NAG-1/GDF15 as a promising therapeutic target for cancer, with potential applications in modulating tumorigenic signaling pathways.

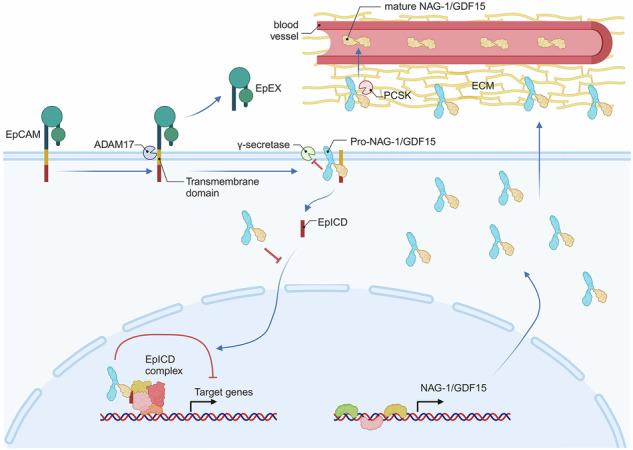

## Introduction

The nonsteroidal anti-inflammatory drug (NSAID)-activated gene-1 (NAG-1), originally identified for its anticancer and anti-inflammatory properties [1], NAG-1 or GDF15 is now recognized as a multifunctional protein involved in various physiological processes such as obesity, aging, and liver diseases. NAG-1/GDF15 has gained attention as a pleiotropic biomarker for several metabolic disorders [2]. It has anti-tumorigenic activity based on the results with mouse models for colorectal cancer [3]. In contrast, elevated levels of NAG-1/GDF15 have been associated with cancer progression, recurrence, and poor survival outcomes in prostate cancer [4]. Therefore, a more thorough understanding of its biological functions and mechanisms is essential to clarify its role in these different conditions.

NAG-1/GDF15 is first synthesized as a pro-form, which is then cleaved to its mature form and secreted into the circulation. The mature form binds to its receptor, GDNF-family receptor α-like (GFRAL), to exert its effects [5]. The pro-NAG-1/GDF15 is also secreted, but in contrast to the mature NAG-1/GDF15, significant levels remain in the cell. Moreover, the pro-NAG-1/GDF15 localizes to the nucleus and influences transcriptional regulation [6], highlighting its potential complex role in gene expression. However, the precise role of the pro-NAG-1/GDF15 form in cancer remains largely unexplored.

EpCAM (Epithelial Cell Adhesion Molecule) is a transmembrane glycoprotein crucial for cell adhesion and the preservation of epithelial tissue integrity. In colorectal cancer, EpCAM is frequently overexpressed, leading to enhanced tumor cell growth, invasion, and metastasis [7]. Due to its high expression levels on the cell surface, EpCAM has become a key target for immunotherapy in colorectal cancer, prompting the development of various therapeutic strategies. The expression of EpCAM is upregulated by 100- to 1000-fold in several primary carcinomas compared to normal tissues [8], underscoring its significance as a cancer stem cell marker [7]. Consequently, investigating the role of EpCAM and its associated secreted proteins within the tumor microenvironment is of considerable interest.

The β-catenin is a pivotal component of the Wnt signaling pathway, and is essential for regulating cellular processes such as proliferation, differentiation, and apoptosis. In colorectal cancer (CRC), mutations in the APC gene or other components of the Wnt pathway frequently result in aberrant β-catenin activation. This dysregulation leads to the accumulation of β-catenin in the nucleus, where it functions as a transcriptional co-activator for genes that promote tumorigenesis [9]. The role of β-catenin in CRC is well-established, particularly in the progression from normal colonic epithelium to adenomas and ultimately invasive carcinoma. Recent studies have advanced our understanding of Wnt/β-catenin signaling in CRC, identifying novel activators and repressors. For instance, the activation of Wnt/β-catenin signaling in EpCAM high/CD44+ cells has been shown to enhance CRC cell proliferation and confer resistance to oxaliplatin treatment [10].

This study highlights the critical roles of NAG-1/GDF15, EpCAM, β-catenin, and NF-κB in the pathophysiology of colorectal cancer (CRC). A deeper understanding of their specific functions and interactions sheds light on the molecular mechanisms driving cancer progression and presents potential opportunities for developing targeted therapies, ultimately aiming to improve patient outcomes.

## Materials and methods

### Cell culture, reagents, and antibodies

Colorectal cancer cell lines HCT116, SW480, CaCo-2, osteosarcoma cell line U2OS, and prostate cancer cell line PC-3 were obtained from the American Type Culture Collection (Manassas, VA, USA), while the LoVo cell line was sourced from the Korean Cell Line Bank (Seoul, Korea). HCT116 and U2OS cells were cultured in McCoy’s 5A medium (Welgene, Gyeongsan, Gyeongsangbuk-do, Korea) supplemented with 10% fetal bovine serum (FBS; Gibco Life Technologies, Carlsbad, CA, USA), 100 U/mL penicillin, and 100 mg/mL streptomycin (Gibco Life Technologies). SW480, CaCo-2, and PC-3 cells were grown in Dulbecco’s Modified Eagle’s Medium (DMEM; Welgene), while LoVo cells were maintained in Roswell Park Memorial Institute (RPMI) 1640 medium (Welgene). All cells were kept in a humidified incubator at 37 °C with 5% CO_2_. All reagents and antibodies used in this study are listed in Supplementary Table 2 and Supplementary Table 3, respectively.

### Stable cell line generation

To create a stable cell line, SW480 cells were transfected with LacZ, NAG-1/GDF15 WT, or NAG-1/GDF15 R193A constructs [6] using PolyJet^TM^ In Vitro DNA Transfection Reagent (SignaGen Laboratories, Frederick, MD, USA) following the manufacturer’s instructions. After transfection, the cells were treated with 1000 μg/ml G418 (InvivoGen, San Diego, CA, USA) every two to three days until all control cells had died. Once established, the SW480 stable cell lines were maintained with 500 μg/ml G418. The HCT116-NAG-1/GDF15-GFP stable cell line, which constitutively expresses NAG-1/GDF15 conjugated to green fluorescent protein (GFP), has been previously described [11].

### Plasmid and siRNA transfection

For transient plasmid transfection, PolyJet^TM^ In Vitro DNA Transfection Reagent (SignaGen Laboratories) was used. For siRNA transfection, PepMute^TM^ siRNA Transfection Reagent (SignaGen Laboratories) was utilized, while PepMute^TM^ Plus siRNA Transfection Reagent (SignaGen Laboratories) was employed for siRNA/DNA co-transfection. All procedures were carried out according to the manufacturer’s protocols. NAG-1/GDF15 siRNA (sc-39798) was obtained from Santa Cruz Biotechnology (Dallas, TX, USA), and control siRNA (SN-1013) was sourced from Bioneer (Daejeon, Korea). The LacZ, NAG-1/GDF15 WT, and NAG-1/GDF15 R193A plasmids have been previously described [6].

### Protein preparation and Western blot analysis

For media concentration, the Pierce^TM^ Protein Concentrator PES (88528, Thermo Fisher Scientific, Waltham, MA, USA) was used according to the manufacturer’s instructions. Protein extraction from the extracellular matrix followed a previously published protocol [12]. Membrane/cytosol fraction was prepared with Mem-PER^TM^ Plus Membrane Protein Extraction Kit (Invitrogen, San Diego, CA, USA) in accordance with the manufacturer’s instructions. Western blot analysis was performed as described in an earlier study [11]. Briefly, cell lysates were separated by sodium dodecyl sulfate-polyacrylamide gel electrophoresis and transferred to nitrocellulose membranes (GVS Filter Technology, Zola Predosa BO, Italy). After antibody labeling, Western blot images were detected and quantified using Alliance Q9 mini (UVITEC, Cambridge, England, UK).

### Co-Immunoprecipitation

Cell lysates for co-immunoprecipitation were prepared using NP-40 lysis buffer (Tris-HCl 50 mM, pH 8.0, NaCl 150 mM, NP-40 1%). After protein concentration quantification with Pierce^TM^ BCA Protein Assay Kits (Invitrogen), 1 mg of protein lysate was incubated with 1 μg of the target antibody overnight at 4 °C with constant rotation. The antigen-antibody complex was then captured using Pierce^TM^ Protein A/G Magnetic Beads (Invitrogen) following the manufacturer’s protocol. Finally, the samples were subjected to immunoblotting for further analysis.

### Surface sensing of translation (SUnSET) assay

To quantify de novo protein synthesis, a SUnSET assay was performed [13]. In brief, puromycin integrates into the protein synthesis machinery upon cell treatment, tagging newly synthesized peptides. Specifically, cells were treated with compounds or transfected with specific plasmids for 24 h, followed by a 30 min incubation with 1 μg/ml puromycin. Cell lysates were then prepared and analyzed by Western blotting. The anti-puromycin antibody was used to detect puromycin-labeled peptides, serving as a marker for de novo protein synthesis.

### Transwell migration and invasion assay

Transwell migration and invasion assays were performed as previously described [14]. Briefly, all stable cell lines were grown to 70–80% confluency, followed by synchronization with serum-free media for 24 h. Next, 1 × 10^5^ SW480 stable cells were seeded onto transwell inserts with or without extracellular matrix coating. Serum-free media was added to the upper chamber, while complete media was added to the lower chamber. After 24 h, unmigrated/uninvaded cells were removed. Remaining cells were fixed with 4% paraformaldehyde and stained with 0.2% crystal violet solution.

### Dual-Luciferase® Reporter assay

Cells were transfected with a designated firefly luciferase plasmid and pRL-null. Details regarding TOPFlash, FOPFlash, pNF-κB-Luc, and pCyclinD1 (−963/+130) plasmids have been previously published [15–17]. In brief, TOPFlash is a luciferase reporter plasmid containing wild-type TCF/LEF binding sites, whereas FOPFlash carries mutant TCF/LEF binding sites. The transcriptional activity of β-catenin was measured by normalizing TOPFlash with FOPFlash. The pNF-κB-Luc contains three copies of NF-κB binding sites, and pCyclinD1 (−963/+130) has one TCF/LEF binding site and three NF-κB binding sites. Cell lysates were harvested, and luciferase activity was measured using the Dual-Luciferase® Reporter Assay System (Promega, Madison, WI, USA) according to the provided protocol. Renilla luciferase activity was used to normalize firefly luciferase activity.

### Inflammasome assay

CaCo-2 cells were transfected with LacZ, NAG-1/GDF15 WT, or NAG-1/GDF15 R193A plasmid, followed by TNF-α treatment for 24 h. An inflammasome assay was performed using the Caspase-Glo® 1 Inflammasome Assay (Promega) according to the manufacturer’s instructions.

### Immunocytochemistry and colocalization analysis

For colocalization between NAG-1 and EpCAM, HCT116-NAG-1/GDF15-GFP was transfected with EpCAM-3XFLAG. After 24 h, cells were labelled with anti-FLAG antibody (Sigma–Aldrich), followed by fixation with 4% paraformaldehyde and nucleus staining with 4′,6-diamidino-2-phenylindole (DAPI; Sigma–Aldrich). Signal colocalization was calculated with CX7 LZR (Thermo Fisher Scientific), and Pearson’s and Mander’s correlation coefficients were used for data analysis.

### Immunofluorescence and signal detection

For EpICD signal detection in the nucleus, HCT116 was transfected with designated plasmids for 24 h. Cells were fixed with 4% paraformaldehyde and stained with DAPI. Fluorescence intensity in the nucleus was measured with CX7 LZR (Thermo Fisher Scientific).

### RNA extraction and quantitative reverse transcription polymerase chain reaction (qRT-PCR)

RNA was extracted from cells or tissue using TRIzol^TM^ Reagent (Thermo Fisher Scientific) or RNeasy Mini Kit (Qiagen, Hilden, Germany), respectively. Complementary DNA was synthesized with Verso cDNA Synthesis Kit (Thermo Fisher Scientific). Quantitative PCR was conducted with PowerUp^TM^ SYBR^TM^ Green Master Mix (Thermo Fisher Scientific) and QuantStudio 1 Real-Time PCR System (Applied Biosystems). All procedures are conducted by the manufacturer’s instructions. For quantification, 2^−ΔΔCt^ method was used with 18S rRNA for normalization. Primers used for qRT-PCR are listed in Supplementary Table 4.

### Phage display screening

To purify mature NAG-1/GDF15, SF9 insect cells were transfected with a baculovirus vector and incubated for five days, after which the media was collected. This media was then used to infect Hi 5 insect cells, which were also grown for five days to achieve a higher concentration of NAG-1/GDF15. The NAG-1/GDF15 was precipitated from the media using 90% ammonium sulfate, followed by centrifugation and dialysis to remove impurities. The purified protein was achieved through Flag-agarose affinity chromatography, with its identity confirmed by SDS-PAGE and western blot analysis. Additionally, a T7 human colon cDNA library (Novagen, CA, USA) was employed in phage display to isolate NAG-1/GDF15-bound phages, which were further analyzed in E. coli using PCR for identification.

### Molecular cloning and site-directed PCR mutagenesis

For EpCAM cloning, pSF-CMV-NEO-COOH-3XFLAG was purchased from Sigma–Aldrich (St. Louis, MO, USA). EpCAM cDNA was synthesized from HCT116. Restriction enzymes HindIII and XhoI (Enzynomics, Daejeon, Korea) were used for molecular cloning. Q5^®^ Site-Directed Mutagenesis Kit (NEB, Ipswich, MA, USA) was used for site-directed PCR mutagenesis according to the provided protocol. Primers used for cloning and site-directed PCR mutagenesis are listed in Supplementary Table 5.

### Animal study

Animal care and procedures were approved by the Institutional Animal Care and Use Committees of Seoul National University (SNU-200922-2-1). Twelve male athymic nude mice were purchased from Koatech (Pyeongtaek, Gyeonggi-do, Korea). Mice were housed in accordance with the College of Veterinary Medicine policies of Seoul National University. Mice were randomly divided into three groups, and 1 × 10^6^ cells were injected into both sides of the thighs. Body weight and tumor size were measured twice every week. After 8 weeks, mice were sacrificed, and xenograft tumors were harvested.

### Immunohistochemistry and TUNEL assay

Immunohistochemistry was performed as previously described [18]. Paraffin-embedded tissue blocks were sectioned and stained with antibodies using the Ultra-Sensitive ABC Staining Kit (Thermo Fisher Scientific) and the ImmPACT® DAB Kit (VECTOR Laboratory, Burlingame, CA, USA), following the manufacturer’s instructions. TACS-XL In Situ Apoptosis Detection Kit (4828-30-DK, R&D Systems, Minneapolis, MN, USA) was used for TUNEL assay in accordance with the manufacturer’s instructions. Images were taken with a microscope Nikon Ti (Nikon, Tokyo, Japan) or a slide scanner Pannoramic SCAN (3DHISTECH, Budapest, Hungary), respectively.

### Public data, in silico protein-protein docking, and statistical analysis

The Cancer Genome Atlas (TCGA) data were analyzed with Gene Expression Profiling Interactive Analysis (GEPIA) [19]. GEPIA is a web-based tool that allows researchers to easily access TCGA data. It was developed to assist those who have difficulty analyzing large datasets from TCGA. By simply typing in a gene name of interest, GEPIA automatically analyzes and provides results from TCGA, such as tumor/normal differential expression analysis, profiling according to cancer types or pathological stages, patient survival analysis, similar gene detection, correlation analysis, and dimensionality reduction analysis. In silico protein-protein docking was simulated with LZerD [20]. An Unpaired Student’s t-test was used for statistical analysis. Graphs represent means ± SD. Data are considered statistically significant at a *p*-value < 0.05. (*p-value < 0.05, **p-value < 0.01, ***p-value < 0.001).

## Results

### Expression of NAG-1/GDF15 in colorectal cancer cell lysates and media

**Fig. 1:**
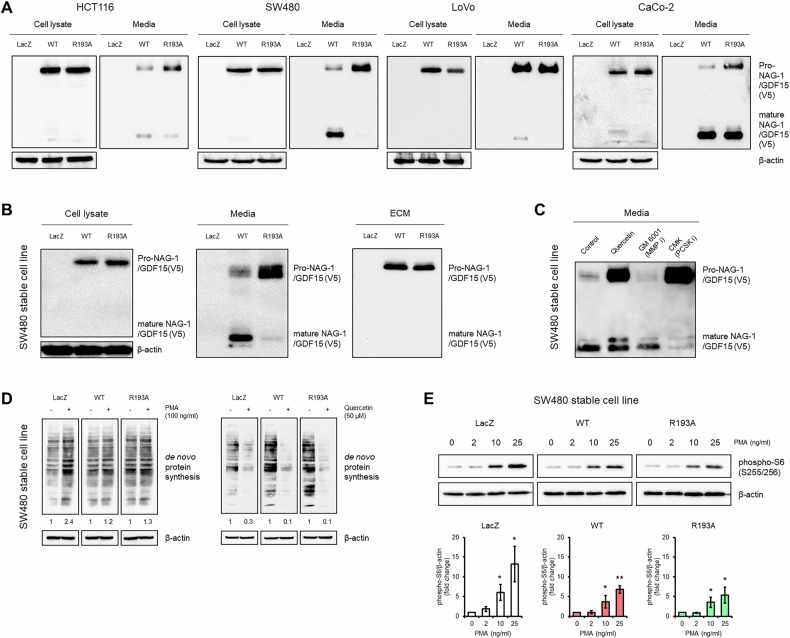
Characteristics of NAG-1/GDF15 overexpressing stable cell lines. **A** NAG-1/GDF15 expression pattern in cell lysate and media from different colorectal cancer cell lines. The expression of pro-NAG-1/GDF15 was higher than that of mature NAG-1/GDF15 in cell lysates. Expression patterns in the media were different between cell lines. **B** SW480 stable cell lines overexpressing LacZ, NAG-1/GDF15 WT, or NAG-1/GDF15 R193A, and their NAG-1/GDF15 expression patterns in cell lysate (left), media (middle), and extracellular matrix (ECM) (right). **C** The NAG-1/GDF15 overexpressing SW480 stable cell line was treated with 50 μM quercetin, 50 μM GM 6001 (pan-MMP inhibitor), 25 μM CMK (pan-PCSK inhibitor) in serum-free media for 24 h, and the ratio between pro- to mature NAG-1/GDF15 was observed. **D** De novo protein synthesis measured after PMA or quercetin treatment in serum-free media for 24 h in SW480 stable cell lines. NAG-1/GDF15 suppresses PMA-induced protein synthesis while enhancing the anticancer activity of quercetin. **E** Phospho-S6 (S255/256) expression with PMA treatment. Stable cell lines were treated with PMA in serum-free media for 24 h. Total cell lysates were subjected to Western blot analysis (top panel). The graphs represent means ± SD (*n* = 3) (bottom panel). *p < 0.05, **p < 0.01.

NAG-1/GDF15 is initially synthesized within cells as pro-NAG-1/GDF15, which undergoes cleavage at the R193 position to produce the mature form and peptide fragment recently named GDPP (GDF15 propeptide) [21]. To clarify the function of pro-NAG-1/GDF15 in cancer cells, we introduced a NAG-1/GDF15 R193A mutant construct that cannot be cleaved into mature NAG-1/GDF15 and GDPP [6]. Figure 1A illustrates the expression patterns of NAG-1/GDF15 within cells and in the media following transfection with either the WT and R193A mutant. Pro-NAG-1/GDF15 was predominantly found inside the colorectal cancer cell lines, while mature NAG-1/GDF15 was minimally or not detectable intracellularly for both the WT and R193A. Interestingly, both the pro-NAG-1/GDF15 and the mature NAG-1/GDF15 were secreted in the media. When comparing the cell lysate and the media, we observed that the ratio of mature NAG-1/GDF15 to pro-NAG-1/GDF15 varied among the different colorectal cancer cell lines. Notably, more mature NAG-1/GDF15 was detected in the media than in the cell lysate across all four colorectal cancer cell lines. Upon transfecting cells with the NAG-1/GDF15 R193A mutant, a decrease in mature NAG-1/GDF15 expression and an increase in pro-NAG-1/GDF15 were observed in the media (Fig. 1A). In conclusion, we found that pro-NAG-1/GDF15 is a major form inside the cell, while mature NAG-1/GDF15 is typically detected in the media.

Next, stable cell lines were generated using SW480 cells, which exhibit low levels of endogenous NAG-1/GDF15 expression compared to other colorectal cancer cell lines used in this study [22]. Three distinct stable cell lines were established, each constitutively expressing either LacZ, NAG-1/GDF15 WT, or the NAG-1/GDF15 R193A plasmid, and these cells were utilized throughout the study. NAG-1/GDF15 expression in the stable cell lines mirrored the results obtained from the transient transfection experiment (Fig. 1B, left and middle panels). Previous research has shown that pro-NAG-1/GDF15 binds to the extracellular matrix (ECM) in prostate cancer cells [23]. To determine if this phenomenon also occurs in colorectal cancer cell line SW480, we analyzed the ECM and observed that pro-NAG-1/GDF15 was present, whereas mature NAG-1/GDF15 was undetectable (Fig. 1B).

Proprotein convertases subtilisin/kexin (PCSK)-3, -5, and -6 have been identified as key enzymes in the cleavage of pro-NAG-1/GDF15 to its mature form [24], and specific Matrix metalloproteinases (MMPs) have also been reported to perform this cleavage [25, 26]. To assess whether inhibiting these enzymes affects the pro-to-mature NAG-1/GDF15 ratio in colorectal cancer cell lines, we treated NAG-1/GDF15 WT stable cell lines with either a pan-MMP inhibitor or a pan-PCSK inhibitor. Treatment with the pan-PCSK inhibitor CMK led to an increase in pro-NAG-1/GDF15 levels, whereas the pan-MMP inhibitor GM 6001 did not produce this effect in colorectal cancer cells (Fig. 1C). Interestingly, we found that quercetin, a phytochemical known for its anticancer properties via NAG-1/GDF15 modulation in various tumor types [27, 28], influenced the pro-to-mature NAG-1/GDF15 ratio. This suggests a potential role for quercetin in regulating the cleavage process of NAG-1/GDF15 in the media. Previous studies have suggested that NAG-1/GDF15 may promote metastasis in colorectal cancer [29], but our findings did not show any increase in migration or invasion activity in SW480 cells (Supplementary Fig. 1).

In our previous work, we showed that NAG-1/GDF15 inhibits TGF-β-induced pro-tumorigenic activity [6] and found that various phytochemicals and NSAIDs exert anti-tumorigenic effects by increasing NAG-1/GDF15 expression [30, 31]. Building on these findings, we explored how NAG-1/GDF15 interacts with different growth factors, carcinogens, and anticancer compounds. Notably, we discovered that both NAG-1/GDF15 WT and NAG-1/GDF15 R193A cell lines affected de novo protein synthesis when treated with PMA or quercetin (Fig. 1D). Furthermore, we detected dose-dependent changes in phospho-S6 expression, a key indicator of mRNA translation, in response to PMA treatment (Fig. 1E). Taken together, we confirmed previous observations on NAG-1/GDF15 expression in various colorectal cancer cells and showed that NAG-1/GDF15 plays a key role in regulating protein synthesis.

### Modulation of β-catenin and NF-κB signaling pathways by NAG-1/GDF15 expression

**Fig. 2:**
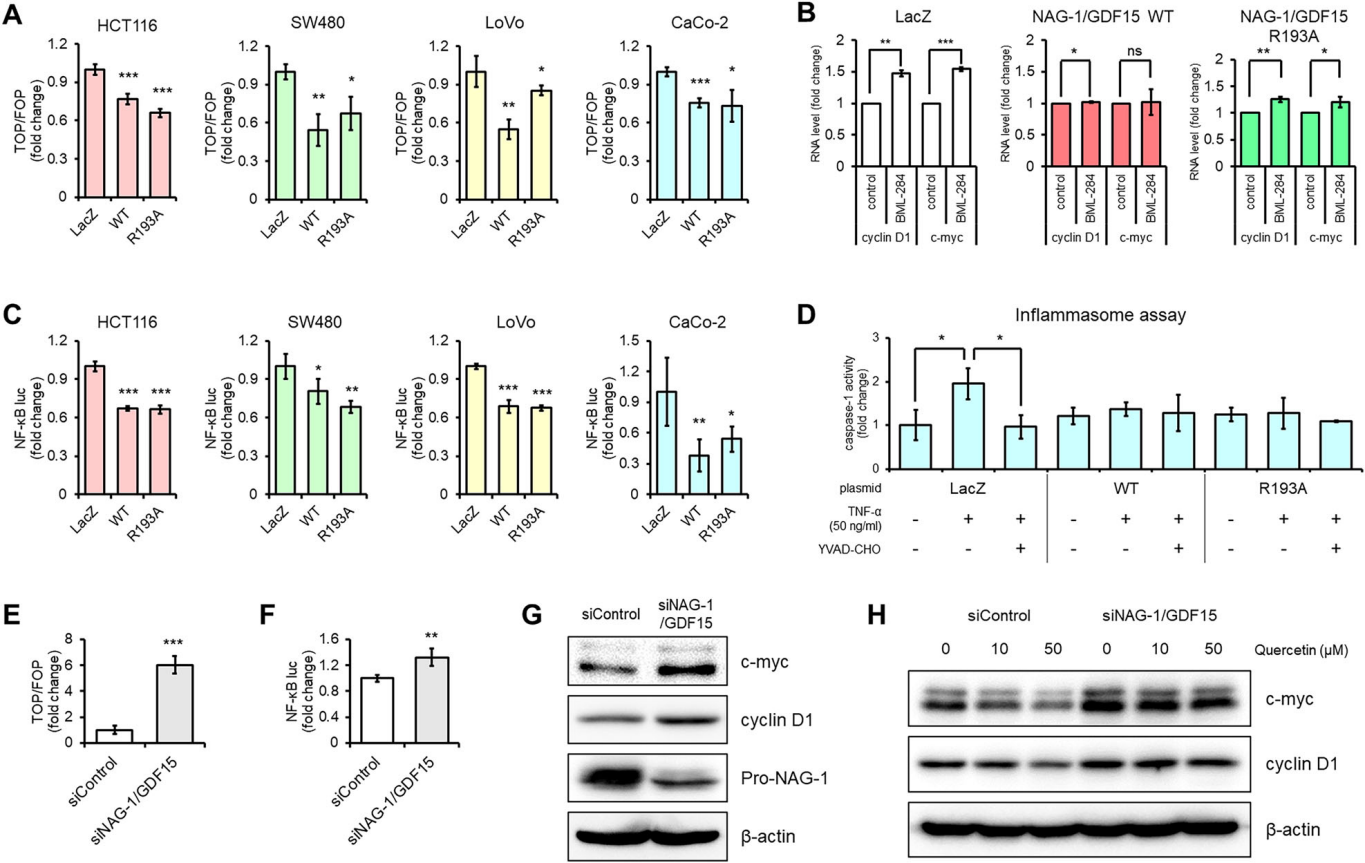
NAG-1/GDF15 blocks β-catenin and NF-κB activity. **A** NAG-1/GDF15 inhibits β-catenin activity in various colon cancer cell lines. (*n* = 4). **B** NAG-1/GDF15 blocks Wnt activator BML-284’s β-catenin target gene induction activity. SW480 stable cell lines were treated with 10 μM BML-284 in serum-free media for 24 h, and the indicated genes were measured by qPCR. (*n* = 3). **C** NAG-1/GDF15 inhibits NF-κB activity in various colon cancer cell lines. (*n* = 4). **D** Inflammasome assay with CaCo-2 cell line treated with TNF-α. TNF-α upregulated caspase-1 activity, while NAG-1/GDF15 WT or R193A overexpression blocked the effect. Caspase-1 inhibitor YVAD-CHO was used to validate the result. (*n* = 3). **E**, **F** Downregulation of NAG-1/GDF15 by siRNA increases β-catenin and NF-κB activity in the HCT116 cell line. (*n* = 4). **G** NAG-1/GDF15 siRNA treatment increases c-myc and cyclin D1 expression in HCT116. Cells were transfected with either control siRNA or NAG-1/GDF15 siRNA for 24 h. **H** Quercetin treatment in serum-free media for 24 h in HCT116 after NAG-1/GDF15 siRNA transfection. NAG-1/GDF15 plays a role in the effect of quercetin downregulating c-myc and cyclin D1. The graphs represent means ± SD. *p < 0.05, **p < 0.01, ***p < 0.001.

NAG-1/GDF15 has been identified as a molecular target for several anticancer compounds, many of which are known to enhance its expression [30, 31]. This prompted us to hypothesize that NAG-1/GDF15’s anticancer effects might be associated with signaling pathways influenced by these compounds. Given that NAG-1/GDF15 inhibited PMA-induced protein synthesis (Fig. 1D), we examined various signaling pathways involved in cancer cell growth. As shown in Fig. 2A, expression of both NAG-1/GDF15 WT and NAG-1/GDF15 R193A significantly suppressed β-catenin activity across different colorectal cancer cell lines. Moreover, when treated with the Wnt activator BML-284, stable cell lines overexpressing NAG-1/GDF15 WT or R193A exhibited a smaller increase in β-catenin target gene expression compared to controls (Fig. 2B).

Beyond the β-catenin pathway, we also observed a reduction in NF-κB activity in colorectal cell lines overexpressing either NAG-1/GDF15 WT or R193A (Fig. 2C). In an inflammasome assay using the CaCo-2 cell line, which showed the strongest response to NAG-1/GDF15 (Fig. 2C), we noted inhibition of caspase-1 activity following TNF-α treatment (Fig. 2D). Besides colorectal cancer cell lines, we found that NAG-1/GDF15 could decrease β-catenin and NF-κB activity in other types of cancer cell lines (Supplementary Fig. 2). To further support our findings that NAG-1 impacts the β-catenin and NF-κB pathways, we used NAG-1/GDF15 siRNA and observed an increase in both β-catenin and NF-κB activity compared to the control (Fig. 2E, F). Additionally, we found upregulation of c-Myc and cyclin D1—both known targets of β-catenin and NF-κB—in cells transfected with NAG-1/GDF15 siRNA (Fig. 2G). To explore NAG-1/GDF15’s role further, we treated cells with quercetin, a compound previously reported to inhibit both the β-catenin and NF-κB signaling pathways [32, 33]. As shown in Fig. 2H, quercetin treatment reduced c-Myc and cyclin D1 expression in the control siRNA-transfected group, but this reduction was less pronounced in cells transfected with NAG-1/GDF15 siRNA. Overall, our findings indicate that both wild-type NAG-1 and the R193 A mutant contribute to the inhibition of β-catenin and NF-κB activity.

### NAG-1/GDF15 interacts with EpCAM via multiple binding sites involving the transmembrane domain and intracellular domain

**Fig. 3:**
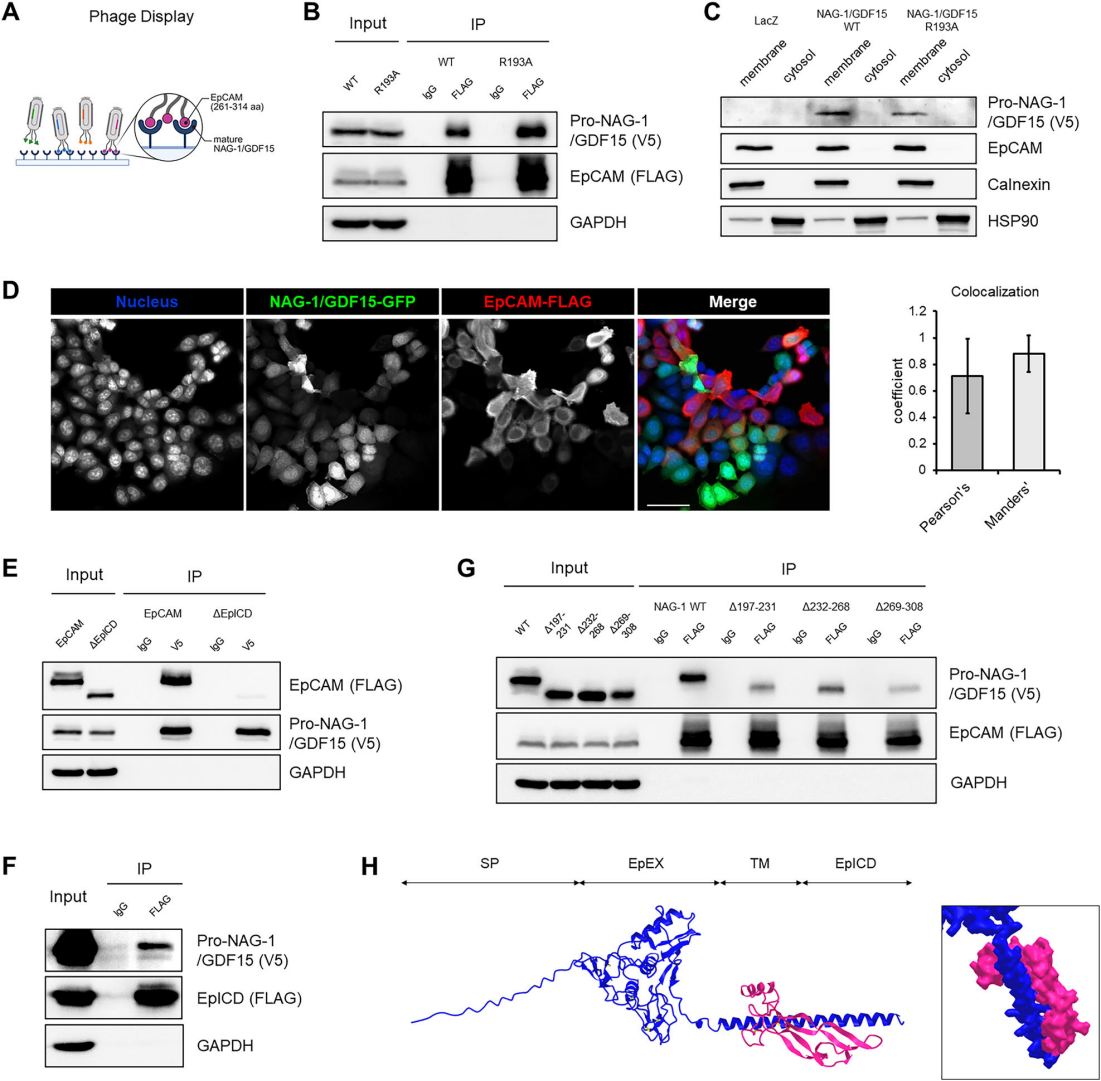
Physical interaction between NAG-1/GDF15 and EpCAM. **A** Phage display screening. Using mature NAG-1/GDF15, potential protein-binding partners for NAG-1/GDF15 were screened. A specific region of EpCAM (261-314 aa) was found to interact with mature NAG-1/GDF15. **B** Validation of phage display data by co-immunoprecipitation. EpCAM was pulled down with an anti-FLAG antibody, and NAG-1/GDF15 WT or R193A were detected with an anti-V5 antibody. **C** Membrane/cytosol fractionation with stable cell lines. NAG-1/GDF15 was found to be expressed in the membrane fraction, along with EpCAM expression. **D** Colocalization of NAG-1/GDF15 and EpCAM. EpCAM was transfected to NAG-1/GDF15-GFP overexpressing HCT116 cells, and Pearson’s or Manders’ coefficient was calculated between NAG-1/GDF15 and EpCAM. Blue: nucleus, Green: NAG-1/GDF15-GFP, Red: EpCAM. Scale bar = 50 μm. The graph represents means ± SD (*n* = 3). **E**, **F** EpICD as the interaction domain for NAG-1/GDF15. By PCR mutagenesis and co-immunoprecipitation, EpCAM’s interaction with NAG-1/GDF15 is abolished when EpICD is deleted, and EpICD itself could bind to NAG-1/GDF15. **G** Serial deletion of NAG-1/GDF15 by PCR mutagenesis, followed by co-immunoprecipitation. Mutated NAG-1/GDF15 constructs showed partial interaction with EpCAM. **H** In silico protein-protein docking. Prediction of NAG-1/GDF15 and EpCAM interaction indicates that mature NAG-1/GDF15 binds EpCAM simultaneously with the transmembrane domain and EpICD. Blue: EpCAM, Red: mature NAG-1/GDF15. SP: signal peptide, EpEX: extracellular domain of EpCAM, TM: transmembrane domain, EpICD: intracellular domain of EpCAM.

To better understand NAG-1/GDF15’s role in inhibiting β-catenin and NF-κB pathways, we conducted phage display screening. Among the potential targets identified (Supplementary Table 1), EpCAM was highlighted as a potential binding partner for NAG-1/GDF15 (Fig. 3A). Since EpCAM is known to promote cancer progression through β-catenin and NF-κB signaling [34, 35], we focused on investigating its interaction with NAG-1/GDF15. Using HCT116, co-immunoprecipitation confirmed that both wild-type NAG-1/GDF15 and the R193A mutant can bind to EpCAM (Fig. 3B). Furthermore, we observed that pro-NAG-1/GDF15, as previously reported [6], is localized in the membrane alongside EpCAM expression in the SW480 stable cell lines (Fig. 3C). We also observed that NAG-1/GDF15 and EpCAM are colocalized using immunofluorescence by transfecting EpCAM in HCT116-NAG-1/GDF15-GFP stable cell line, based on Pearson’s and Mander’s correlation coefficient calculation (Fig. 3D). Next, we aimed to pinpoint the specific regions of NAG-1/GDF15 and EpCAM that are responsible for their interaction. Guided by our phage display findings, we focused on the EpCAM intracellular domain (EpICD). To explore its role, we used PCR mutagenesis to remove the EpICD from EpCAM, which resulted in a significant reduction in the binding between NAG-1/GDF15 and EpCAM (Fig. 3E). This suggests that EpICD is crucial for the interaction. Interestingly, we confirmed that EpICD alone is sufficient to bind NAG-1/GDF15 (Fig. 3F), reinforcing its key role in mediating the interaction between these two proteins. Next, we performed serial deletions on NAG-1/GDF15 to determine which regions are crucial for its interaction with EpCAM. We anticipated that removing key binding regions would cause the interaction to disappear, similar to what we observed in Fig. 3E. However, instead of a complete loss, we observed only partial reductions in binding across all deletion groups (Fig. 3G). To further investigate these results, we employed in silico protein-protein docking analysis. The analysis revealed that, unlike typical protein-protein interactions where a single binding site is critical, NAG-1/GDF15 appears to interact with EpCAM simultaneously across both the transmembrane domain and the EpICD (Fig. 3H). Overall, our findings confirm that NAG-1/GDF15 binds to EpCAM through multiple contact points.

### NAG-1/GDF15 inhibits EpCAM activity by blocking EpCAM cleavage and nuclear entry of EpICD

**Fig. 4:**
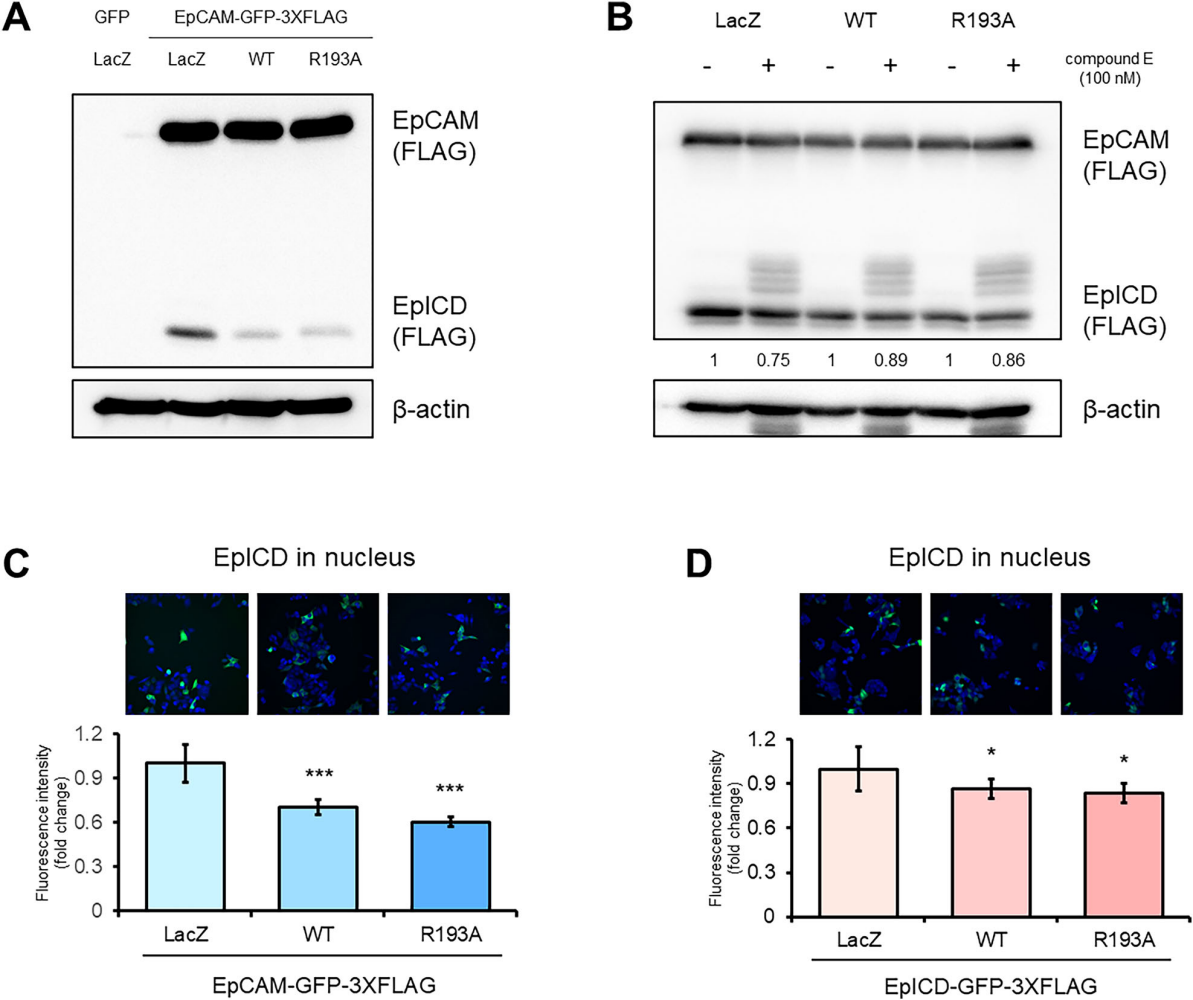
NAG-1/GDF15 inhibits EpCAM cleavage and EpICD translocation to the nucleus. **A** Inhibition of EpCAM cleavage to EpICD by NAG-1/GDF15 WT and R193A. HCT116 cells were transfected as indicated, and expression of EpICD was observed by Western blotting. **B** HCT116 cells were transfected with EpCAM and subsequently treated with Compound E, a well-known γ-secretase inhibitor. Total cell lysates were analyzed by Western blot, and the relative levels of EpICD are shown at the bottom. **C**, **D** EpICD expression in the nucleus was evaluated. HCT116 cells were transfected with the indicated plasmid along with either EpCAM-GFP-3XFLAG or EpICD-GFP-3XFLAG, and nuclear EpICD expression was assessed by measuring green fluorescence intensity. Blue indicates the nucleus, and green indicates EpICD (*n* = 6). The graphs represent means ± SD. *p < 0.05, ***p < 0.001.

We next explored whether NAG-1/GDF15’s interaction with EpCAM affects EpCAM’s activity. As shown in Fig. 4A, pro-NAG-1/GDF15 was found to inhibit the cleavage of EpCAM into its intracellular domain (EpICD). Since EpCAM cleavage is mediated by γ-secretase [34], we treated cells with the γ-secretase inhibitor Compound E. In HCT116 cells overexpressing either NAG-1/GDF15 WT or the R193A mutant, we observed only a slight change, indicating that NAG-1/GDF15 may partially inhibit γ-secretase activity (Fig. 4B). To explore this, we examined EpICD levels in the nucleus and found that its presence was reduced when NAG-1/GDF15 was overexpressed (Fig. 4C). This led us to hypothesize that NAG-1/GDF15 not only inhibits EpCAM cleavage but also prevents EpICD from entering the nucleus. To test this, we overexpressed EpICD in HCT116 cells and observed that, despite increased levels of EpICD, its nuclear localization was still reduced in the presence of NAG-1 (Fig. 4D). Overall, our findings suggest that NAG-1/GDF15, through its interaction with EpCAM, inhibits both EpCAM cleavage and the nuclear translocation of EpICD.

### NAG-1/GDF15 inhibits EpCAM oncogenic activity by suppressing β-catenin and NF-κB pathways

**Fig. 5:**
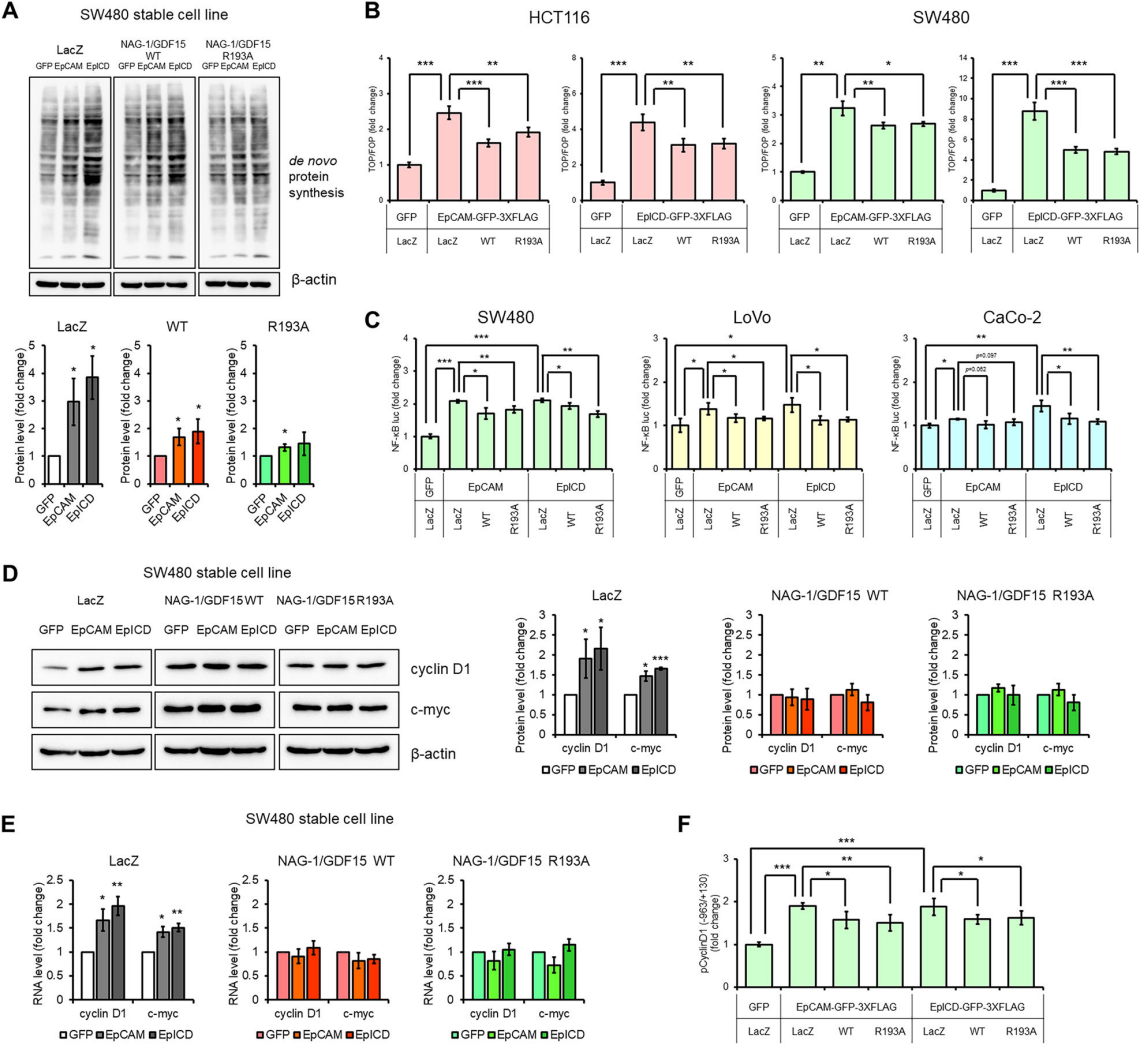
NAG-1/GDF15 blocks oncogenic EpCAM activity. **A** De novo protein synthesis measurement after EpCAM or EpICD transfection in SW480 stable cell lines. Stable cell lines were transfected with EpCAM or EpICD, followed by puromycin treatment. Newly synthesized proteins were measured by Western blotting (top panel). NAG-1/GDF15 WT and R193A overexpressing stable cell lines showed a smaller increase compared to the control. The data was quantified and represented as graphs (bottom panel). (*n* = 3). **B** NAG-1/GDF15 inhibits EpCAM-induced β-catenin activity. (*n* = 4). **C** NAG-1/GDF15 inhibits EpCAM-induced NF-κB activity. (*n* = 3). **D** NAG-1/GDF15 suppresses EpCAM-induced c-myc and cyclin D1 protein expression. Representative images of cyclin D1 and c-myc Western blot (left panel) and quantification (right panel). (*n* = 3). **E** NAG-1/GDF15 suppresses EpCAM-induced c-myc and cyclin D1 RNA expression. (*n* = 3). **F** NAG-1/GDF15 reduces EpCAM-induced cyclin D1 promoter activity. SW480 cells were transfected with the pCyclinD1 construct, containing both TCF/LEF and NF-κB response elements, and luciferase activity was subsequently measured. (*n* = 4). The graphs represent means ± SD. *p < 0.05, **p < 0.01, ***p < 0.001.

We then explored the molecular mechanisms by which NAG-1/GDF15 inhibits the oncogenic activity of EpCAM. As shown in Fig. 5A, both EpCAM and EpICD expression enhanced de novo protein synthesis, but this effect was reduced in cell lines overexpressing either NAG-1/GDF15 WT or NAG-1/GDF15 R193A. Additionally, while EpCAM and EpICD increased β-catenin activity, NAG-1/GDF15 effectively counteracted this increase (Fig. 5B). Moreover, NAG-1/GDF15 inhibited NF-κB activity induced by EpCAM and EpICD in SW480 and LoVo colorectal cell lines, and specifically blocked EpICD-induced NF-κB activity in CaCo-2 cells (Fig. 5C). To evaluate the impact of NAG-1/GDF15 expression on β-catenin and NF-κB target genes, we measured levels of cyclin D1 and c-myc. Expression of both NAG-1/GDF15 WT and R193A resulted in reduced levels of these target genes compared to the LacZ-transfected control (Fig. 5D). Further analysis of RNA levels indicated that NAG-1/GDF15 modulates EpCAM-induced expression of cyclin D1 and c-myc (Fig. 5E). Finally, using a luciferase reporter construct with β-catenin and NF-κB binding sites, we found that overexpression of NAG-1/GDF15 WT and R193A reduced the effect of EpCAM and EpICD on cyclin D1 promoter activity (Fig. 5F). Thus, NAG-1/GDF15 seems to inhibit the oncogenic activity of EpCAM, at least partially, by suppressing β-catenin and NF-κB activity, potentially acting as a co-repressor in these pathways.

### NAG-1/GDF15 expression reduces tumor growth in colorectal cancer xenograft model

**Fig. 6:**
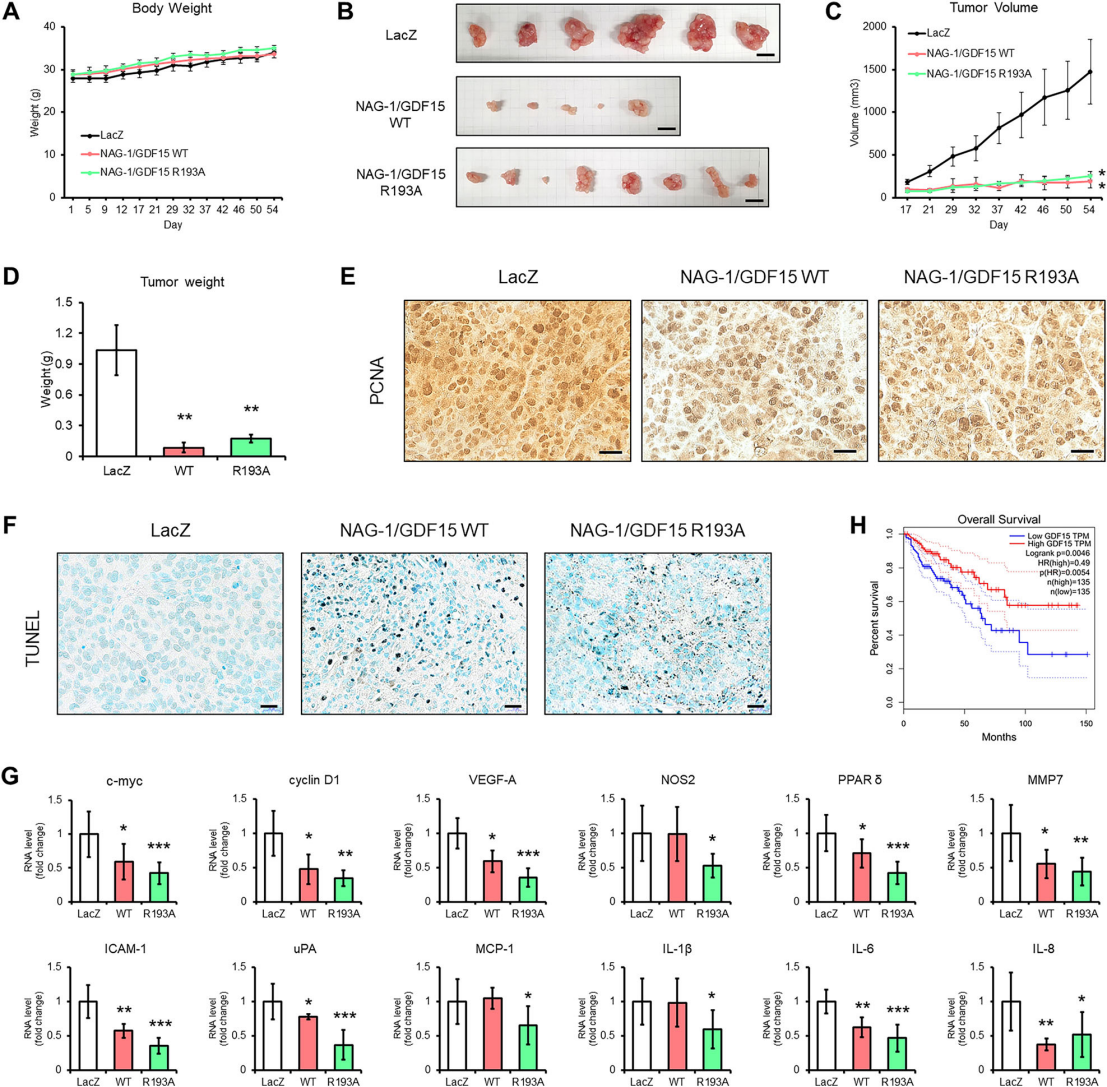
NAG-1/GDF15 suppresses colorectal cancer growth in vivo. **A** Nude mice body weight. **B** Representative images of xenograft tumors. **C**, **D** Tumor volume and weight. **E** Immunohistochemistry of PCNA with xenograft tumors. NAG-1/GDF15 WT and R193A showed less PCNA expression compared to the control. Scale bar = 25 μm. **F** TUNEL assay. NAG-1/GDF15 WT and R193A tumors showed increased DNA breaks compared to the control. Scale bar = 20 μm. **G** β-catenin and NF-κB target genes expression in xenograft tumors. NAG-1/GDF15 WT and R193A expression decreased target gene expression. (LacZ *n* = 6, NAG-1/GDF15 WT *n* = 4, NAG-1/GDF15 R193A *n* = 7). **H** Kaplan–Meier curve of colorectal cancer patients regarding NAG-1/GDF15. Data extracted from TCGA (https://www.cancer.gov/ccg/research/genome-sequencing/tcga). The high NAG-1/GDF15 group showed better survival compared to the low NAG-1/GDF15 group. The graphs represent means ± SD. *p < 0.05, **p < 0.01, ***p < 0.001.

Using the SW480 stable cell line, we performed an in vivo xenograft model to assess the effects of NAG-1/GDF15 expression on tumor development. Although we anticipated a decrease in body weight in NAG-1/GDF15 WT overexpressing tumor-bearing nude mice given that mature NAG-1/GDF15 is known to reduce body weight through binding to GFRAL in the brain [5], but no significant differences in body weight were observed between the groups (Fig. 6A). However, post-experiment analysis revealed that the NAG-1/GDF15 WT and NAG-1/GDF15 R193A groups exhibited smaller tumor size, volume, and weight, with less redness compared to the control group (Fig. 6B, C, D). Immunohistochemical analysis demonstrated a reduction in PCNA expression, a marker of cell proliferation, in both the NAG-1/GDF15 WT and R193A groups (Fig. 6E). Additionally, the TUNEL assay showed an increase in DNA fragmentation, suggesting higher levels of cell death in these groups compared to the control (Fig. 6F). Further analysis of β-catenin and NF-κB target genes showed a decrease in their expression with NAG-1/GDF15 overexpression in colorectal cancer cells (Fig. 6G). Notably, changes in RNA levels of NOS2, MCP-1, and IL-1β were observed only in the R193A group. Additionally, data from public databases revealed that colorectal cancer patients with high NAG-1/GDF15 expression had better survival rates compared to those with low NAG-1 expression, as shown by TCGA data (Fig. 6H). Although a similar trend was noted in rectal cancer, the results were not statistically significant (Supplementary Fig. 3). Taken together, NAG-1/GDF15 effectively inhibits colorectal cancer growth in vivo.

## Discussion

NAG-1/GDF15, a member of the TGF-β superfamily, exhibits biological activity that varies depending on the context. Similar to TGF-β, which has been observed to have both pro-cancer and anticancer effects [36], research on NAG-1/GDF15 has produced conflicting results regarding its role in cancer. For instance, NAG-1/GDF15 has been implicated in the progression of prostate cancer, as evidenced by its role in promoting bone metastasis in prostate cancer cells [37]. Additionally, elevated serum levels of NAG-1/GDF15 in prostate cancer patients are associated with reduced survival rates [4]. In contrast, others have extensively studied NAG-1/GDF15 as a tumor suppressor gene in colorectal cancer [1, 3, 6, 11]. For example, the expression of NAG-1/GDF15 in a standard mouse model (*Apc-Min*) for intestinal cancer suppressed the formation of intestinal neoplasia, providing evidence for in vivo tumor suppressor activity [6]. In addition, colon cancer patients with high NAG-1/GDF15 levels exhibit longer survival, according to our analysis of TCGA data (Fig. 6H). This led us to investigate the specific mechanisms behind its anticancer activity in colorectal cancer.

NAG-1/GDF15 is synthesized initially as a pro-NAG-1/GDF15 precursor, which is then cleaved into its mature form. Unlike in prostate cancer cell lines, we observed that in colorectal cancer cell lysates, only pro-NAG-1/GDF15 is highly expressed, with little to no detection of mature NAG-1/GDF15 (Fig. 1A). We hypothesized that these distinct expression patterns of NAG-1/GDF15 forms within cells could lead to the contradictory roles of NAG-1/GDF15 in cancer. To investigate this issue, we utilized a plasmid construct that exclusively expresses pro-NAG-1/GDF15 (NAG-1 R193A). NAG-1/GDF15 contains a cleavage site, “RXXR,” at the 190–193 amino acid position. However, another RXXR site exists at positions 209–212, which may also be cleaved by enzymes. Interestingly, unlike other colorectal cancer cells, Caco-2 cells appear to utilize this second site, as the NAG-1/GDF15 R193A construct did not prevent the production of the mature form (Fig. 1A). Although further analysis of NAG-1/GDF15 processing mechanism in CaCo-2 cells is required, it is interesting to know that the second site could be used in a cell specific manner.

In our previous research, we demonstrated that NAG-1/GDF15 can inhibit TGF-β-induced tumorigenesis [6]. Building on this, we hypothesized that NAG-1/GDF15 might also resist external stimuli that promote cell proliferation, leading us to screen various carcinogens and cytokines. Our findings confirmed that NAG-1/GDF15 can indeed block PMA-induced protein synthesis (Fig. 1D, E) and inhibit the effects of the Wnt activator BML-284 (Fig. 2B). Additionally, NAG-1/GDF15 expression was shown to block TNF-α-induced inflammasome activity (Fig. 2D). NAG-1/GDF15’s ability to counteract growth factors and cytokines may help explain the results of our xenograft experiments, where NAG-1/GDF15 WT and R193A stable cell lines exhibited significant differences compared to the control. This suggests that NAG-1/GDF15 could effectively neutralize growth factors and cytokines released from the tumor microenvironment. This study also shows that pro-NAG-1/GDF15 alone can exhibit anticancer activity.

We have previously reported that various phytochemicals and NSAIDs appear to have anticancer effects through the induction of NAG-1/GDF15 expression [30, 31]. Based on this, we hypothesized that NAG-1/GDF15 might enhance the effects of anticancer compounds, or even be essential for their activity. Our results showed that NAG-1/GDF15 enhances the effects of quercetin, a polyphenol known for its anti-tumor properties (Fig. 1D), while silencing NAG-1/GDF15 with siRNA reduced quercetin’s efficacy (Fig. 2H). Interestingly, we also observed a similar shift in the ratio between pro-NAG-1/GDF15 and mature NAG-1/GDF15, when cells were treated with quercetin (Fig. 1C), a pattern similar to that seen with CMK, a pan-PCSK inhibitor that blocks NAG-1/GDF15 maturation. This suggests that quercetin may act as a PCSK inhibitor to exert its anticancer effects, aligning with recent recognition of PCSK inhibitors as a novel therapeutic approach for cancer treatment [38, 39]. Furthermore, a recent study indicates that NSAIDs reduce inflammation via NAG-1/GDF15 expression in vivo [40]. Our group also published findings showing that sulindac and tolfenamic acid can prevent the development of intestinal neoplasia through NAG-1/GDF15 expression [41, 42]. The fact that NAG-1 is readily induced by phytochemicals and NSAIDs, highlights its potential as a promising target for the development of anticancer drugs.

We demonstrated that NAG-1/GDF15 affected de novo protein synthesis when cells were treated with PMA or quercetin (Fig. 1). Given that mRNA translation is closely linked to cancer cell growth [43], and NAG-1/GDF15 is induced by anticancer compounds affecting various signaling pathways, we focused on investigating whether NAG-1/GDF15 can inhibit growth-related signal transduction. Through screening multiple signaling pathways, we identified β-catenin and NF-κB signaling as promising targets for NAG-1/GDF15’s anticancer effects (Fig. 2). Wnt/β-catenin signaling is well-known in colorectal cancer, with mutations or abnormal activity frequently contributing to its development [9]. Similarly, aberrant activation of NF-κB is commonly observed in colorectal cancer, making it a key focus for therapeutic intervention [44]. In this study, we showed that upregulation of NAG-1/GDF15 decreases, while downregulation of NAG-1/GDF15 increases β-catenin and NF-κB activity in colon cancer cell lines (Fig. 2). The tumor suppression activity of NAG-1/GDF15 and pro-NAG-1/GDF15 was directly examined with a xenograft mouse model. Both NAG-1/GDF15 and pro-NAG-1/GDF15 greatly reduced tumor size. Furthermore, tumors derived from both NAG-1/GDF15 and pro-NAG-1/GDF15 expressing cells exhibited reduced β-catenin and NF-κB activity, as evidenced by the decreased expression of β-catenin and NF-κB target genes in vivo following NAG-1/GDF15 expression (Fig. 6G).

We found that some genes were only changed in the R193A stable cell line. This may result from that mature NAG-1/GDF15 provokes cancer-surrounding cells like macrophages or endothelial cells to produce cytokines, in turn inducing cancer growth. This result reinforces our hypothesis that NAG-1/GDF15 exerts anticancer activity as a pro-form inside the cells, while inducing cancer proliferation when it exists as a mature form outside the cells. Indeed, a recent study reports that the propeptide domain of pro-NAG-1/GDF15, which remains after the cleavage of mature NAG-1/GDF15, promotes proliferation, invasion, and migration in prostate cancer cell lines when administered as a treatment [21]. On the other hand, our data suggest that pro-NAG-1/GDF15 overexpression in prostate cancer cells exerts anticancer activity (Supplementary Fig. 2). There is a possibility that secreted pro-NAG-1/GDF15 shows pro-tumorigenic activity; however, the treated pro-NAG-1/GDF15 could undergo maturation, thereby producing the oncogenic mature NAG-1/GDF15 and propeptides (GDPP). Thus, cleavage at the RXXR site is crucial in determining whether NAG-1/GDF15 exhibits anti- or pro-tumor activity. While additional data is necessary to confirm this hypothesis, it provides a framework for understanding the complex and contradictory biological functions reported for NAG-1/GDF15. Since prostate cancer highly expresses PCSKs more than other types of cancers [21], our hypothesis might explain the discrepancy in prognosis between prostate cancer and colorectal cancer patients. Prostate cancer patients with high NAG-1/GDF15 exhibit poor prognosis [4], while colorectal cancer patients show the opposite trend (Fig. 6H). Altogether, these findings suggest that NAG-1/GDF15 could be an effective molecular target for colorectal cancer treatment, as it simultaneously impacts multiple signaling pathways, potentially generating synergistic anticancer effects.

Recent studies have identified potential protein interaction partners for NAG-1/GDF15. For example, mature NAG-1/GDF15 binds to GFRAL in the brain, leading to reduced appetite and body weight [5]. Glucuronyl C5-epimerase interacts with mature NAG-1/GDF15 to prevent its degradation and thereby exerts increasing anti-obesity effects [45]. Building on these findings, we investigated protein interactions with NAG-1/GDF15 to better understand the mechanisms behind its suppression of β-catenin and NF-κB activity. Through phage display screening, we identified several candidate proteins (Supplementary Table 1). Among these, we focused on EpCAM, a protein previously reported to upregulate β-catenin and NF-κB signaling [34, 35]. EpCAM, initially identified in colorectal cancer cell lines [46], is a membrane-bound protein with oncogenic properties. It is highly expressed in colorectal cancer and is known to promote proliferation and metastasis [7]. Elevated levels of EpCAM are associated with poor prognosis in colorectal cancer patients [47]. EpCAM undergoes cleavage by ADAM17, producing EpEx (the extracellular domain), which is further processed by γ-secretase to generate EpICD (the intracellular domain). EpEx contains an EGF-like domain and acts as a ligand to stimulate cell proliferation [48], while the cleaved EpICD enters the nucleus and regulates β-catenin signaling [34]. Beyond β-catenin, EpCAM also influences other signaling pathways, including NF-κB [35], AP-1 [49], PI3K/Akt/mTOR [50], Ras [51], EGFR [52], and HGFR [53] pathways.

In this study, we discovered that NAG-1/GDF15 can bind to EpCAM (Fig. 3). We propose that this interaction blocks the cleavage of EpCAM into EpICD, thereby preventing EpICD from entering the nucleus (Fig. 4). Several mechanisms could explain this phenomenon. First, the interaction with NAG-1/GDF15 might prevent EpCAM from reaching the membrane after synthesis. Alternatively, NAG-1/GDF15 could physically obstruct the cleavage site of EpCAM, hindering γ-secretase from processing it into EpICD. Additionally, the interaction between NAG-1/GDF15 and EpICD might cover the nuclear localization signal of EpICD, thereby interfering with its nuclear entry. One of the components of this complex is FHL2, which directly binds to EpICD. There is a report suggesting that phosphorylation of Tyr297 on EpICD may be important for stabilizing its α-helical structure, which enhances its binding with FHL2 [54]. Our in silico analysis indicates that NAG-1/GDF15 interacts with EpICD in a manner that encases and potentially shields the Tyr297 site (Fig. 3H). This interaction may interfere with the binding of EpICD to FHL2, thereby downregulating their activity. Moreover, we found that NAG-1/GDF15 can reverse EpCAM-induced β-catenin and NF-κB activity (Fig. 5). This effect may result from NAG-1/GDF15’s ability to physically inhibit EpCAM, but it is also possible that NAG-1/GDF15 functions as a co-repressor. Supporting this idea, we observed that NAG-1/GDF15 can bind to β-catenin (Supplementary Fig. 4). We previously reported that pro-NAG-1/GDF15 can enter the nucleus and inhibit the Smad pathway in a transcription-dependent manner [6]. Similarly, pro-NAG-1/GDF15 might regulate transcription initiated by EpICD in the nucleus. Further research is needed to elucidate the specific mechanisms by which pro-NAG-1/GDF15 inhibits EpCAM.

In conclusion, our study shows that NAG-1/GDF15, via its full-length pro-NAG-1/GDF15 form, functions intracellularly as a tumor suppressor by inhibiting the oncogenic protein EpCAM, which in turn reduces β-catenin and NF-κB signaling. These findings align with the reduced inflammatory response observed in transgenic mice expressing NAG-1/GDF15 [55], where tumor suppressor activity was also evident.

## Supplementary information

Original WB file

Supplementary figure (1,2,3, 4) and Tables (1, 2, 3–1, 3–2, 3–3, 4, 5)

## Data Availability

All data generated or analyzed during this study are included in this published article and its supplementary information files.
