# Supplementary material for: NAG-1/GDF15 as a tumor suppressor in colorectal cancer: inhibition of β-catenin and NF-κB pathways via interaction with EpCAM
Source: Cell Death Dis. 2025 May 2;16(1):355. doi: 10.1038/s41419-025-07695-w (PMC12048721; doi:10.1038/s41419-025-07695-w)

Figure 1

A

HCT116

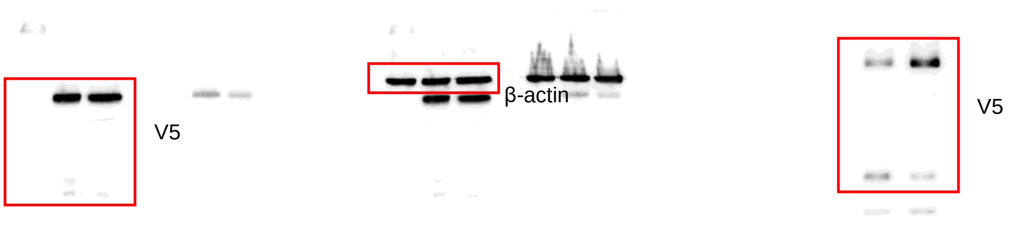

SW480

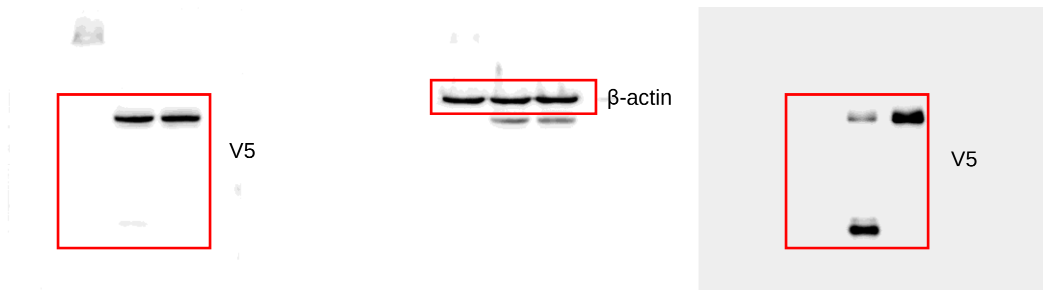

LoVo

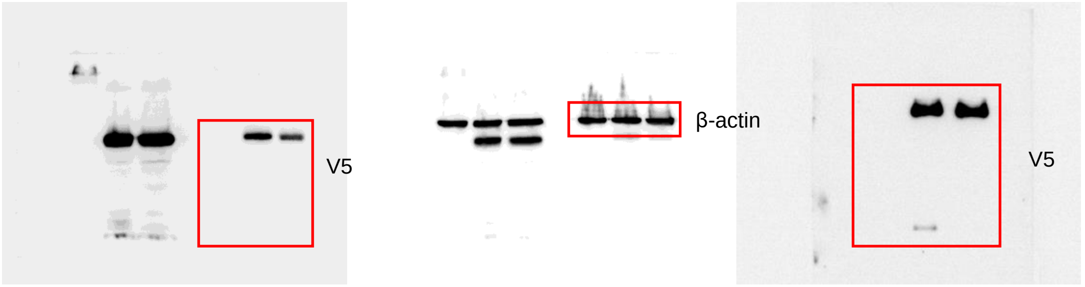

CaCo-2

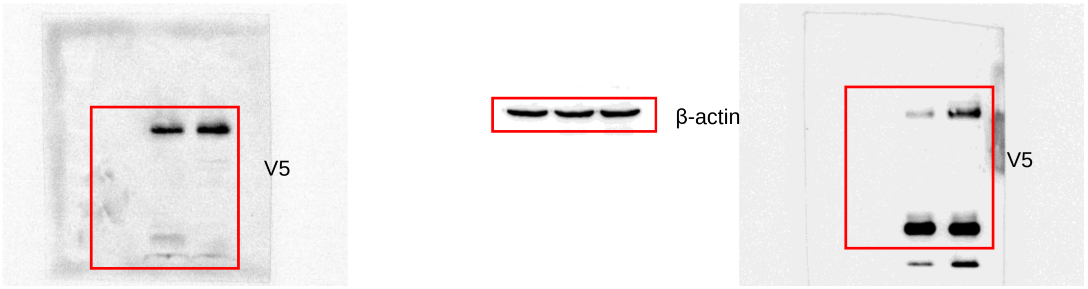

Figure 1

B

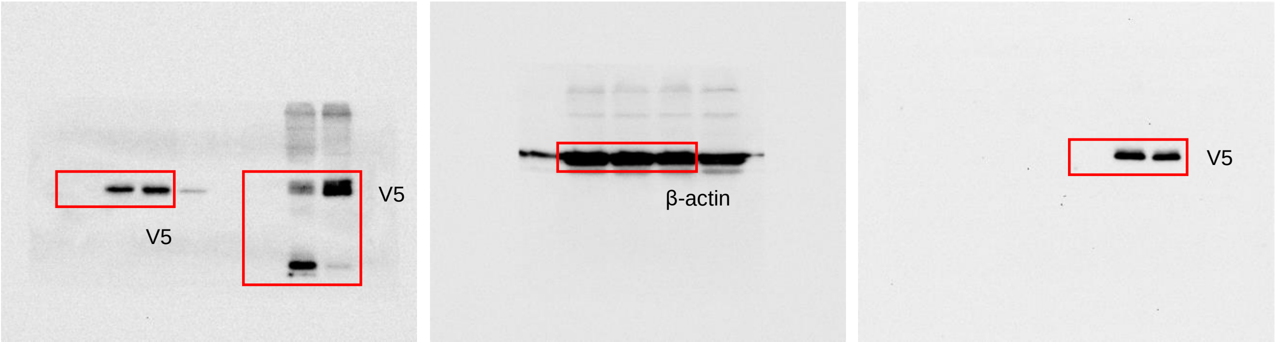

C

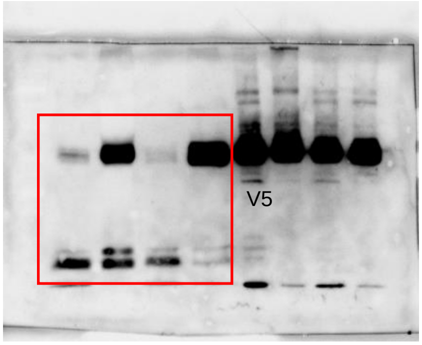

D

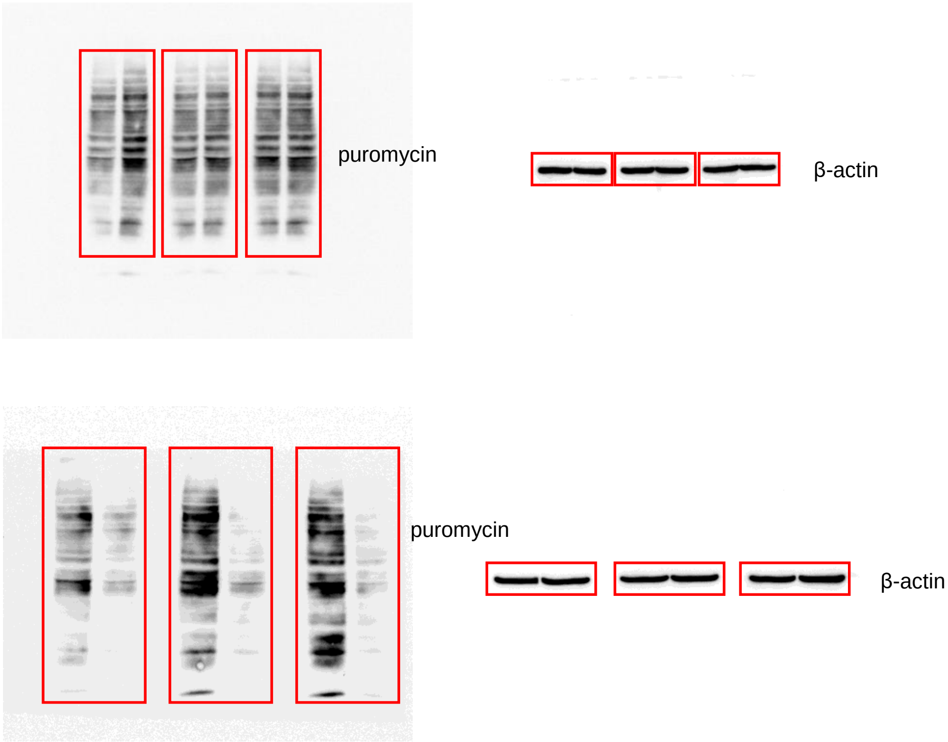

Figure 1

E

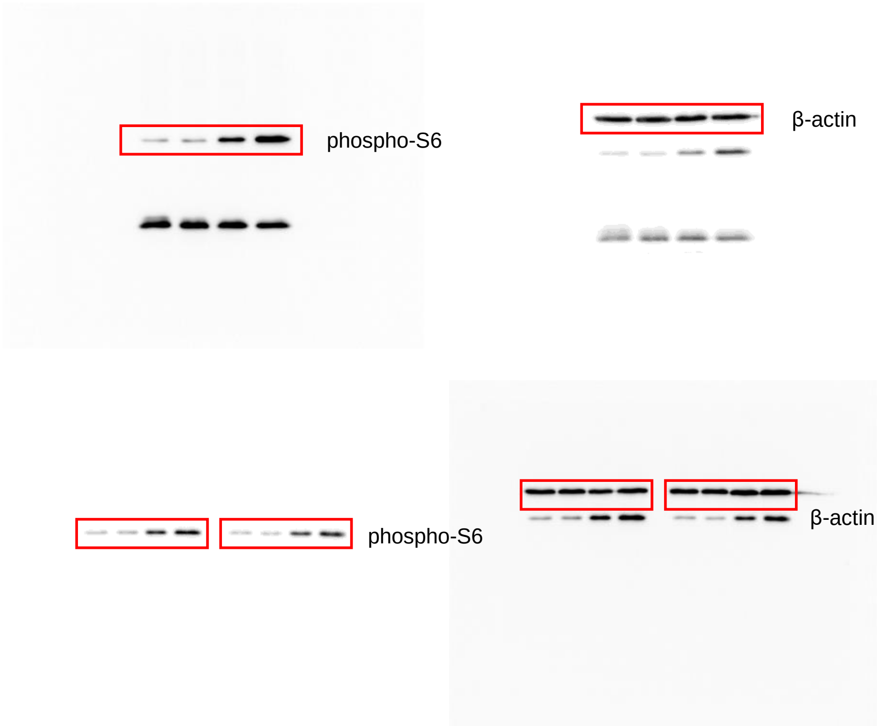

Figure 2

G

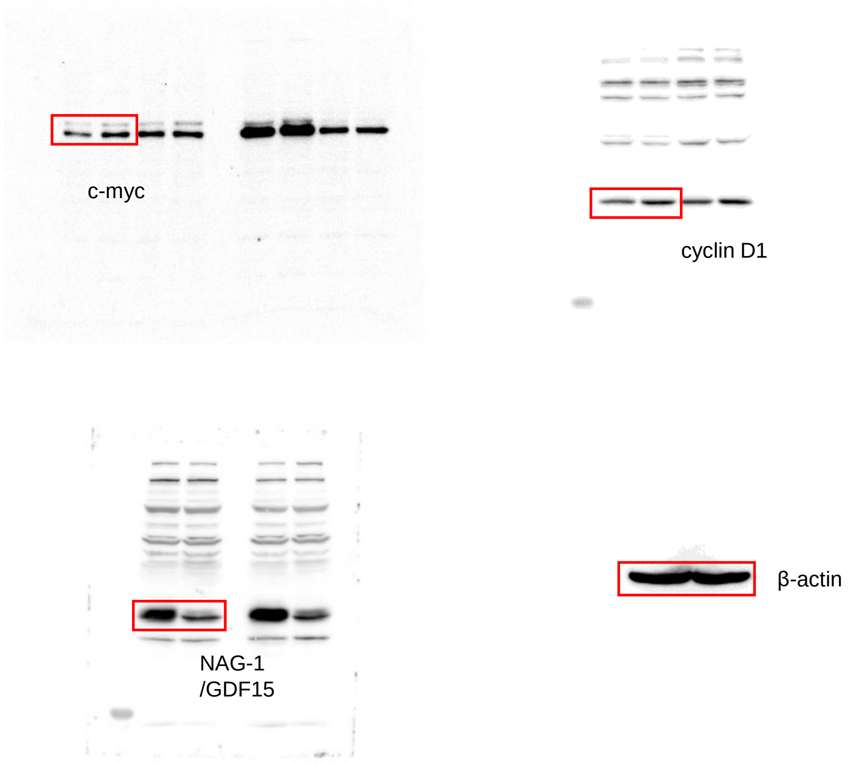

Figure 2

H

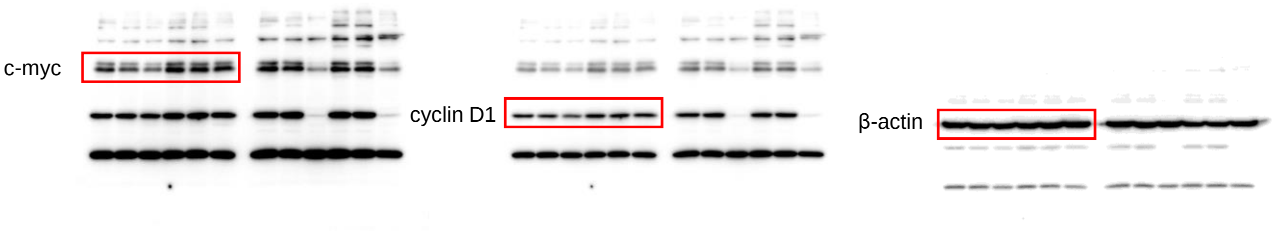

Figure 3

B

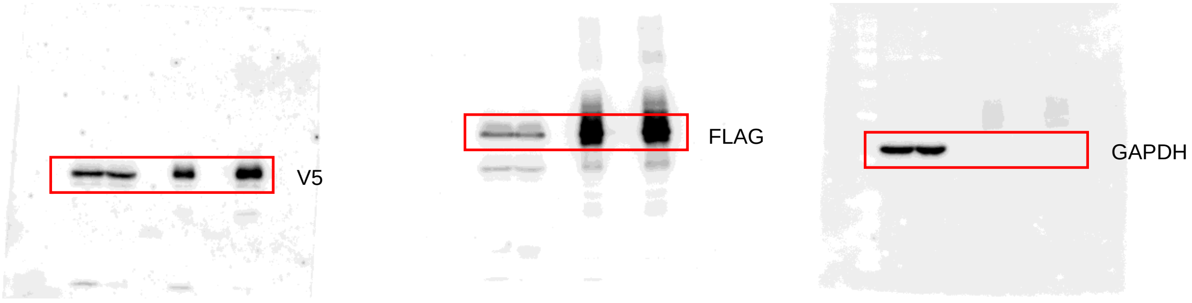

C

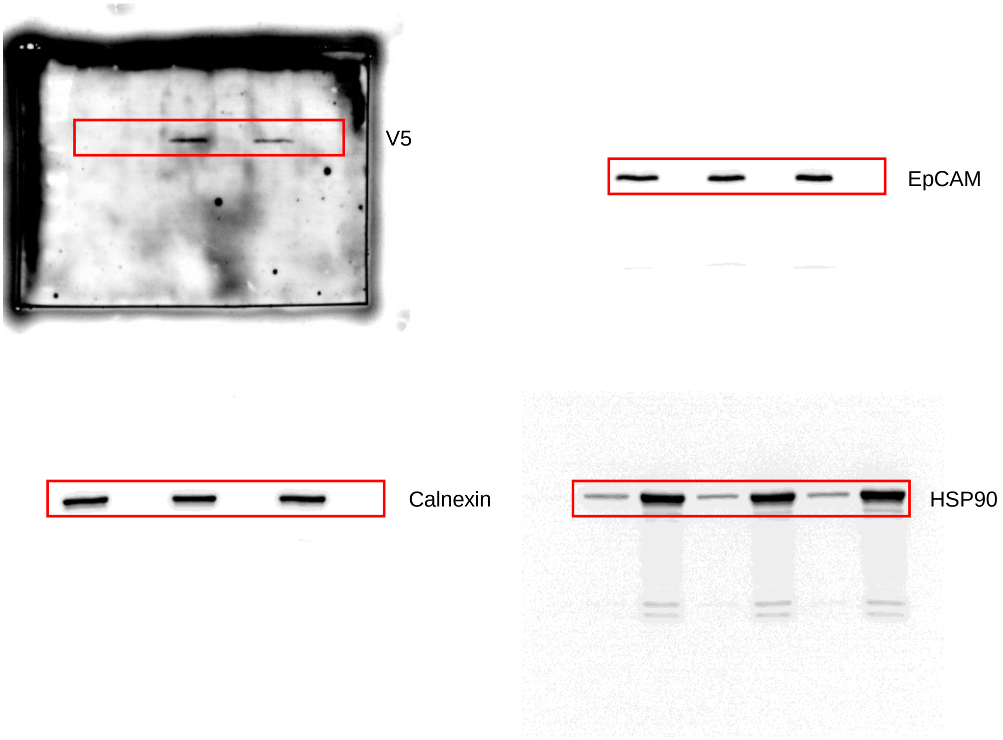

Figure 3

E

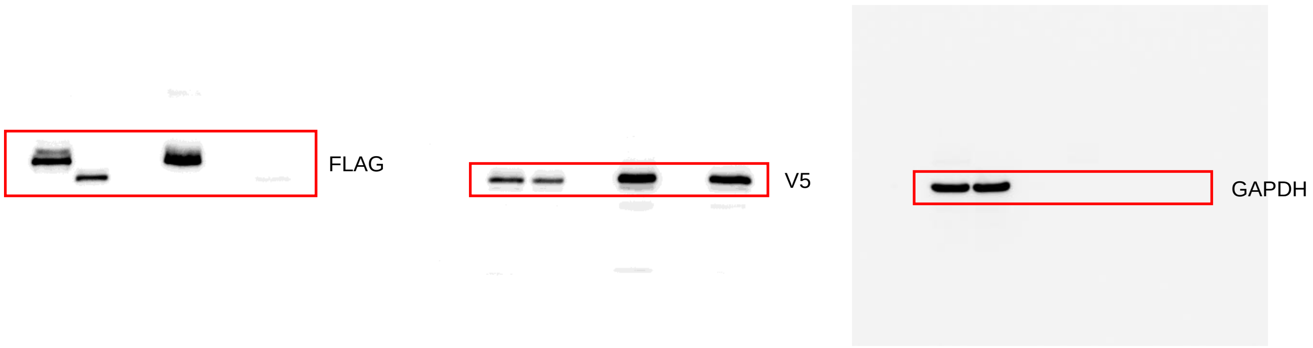

F

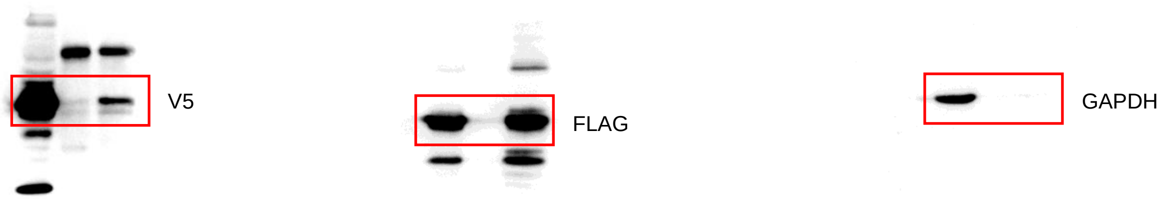

G

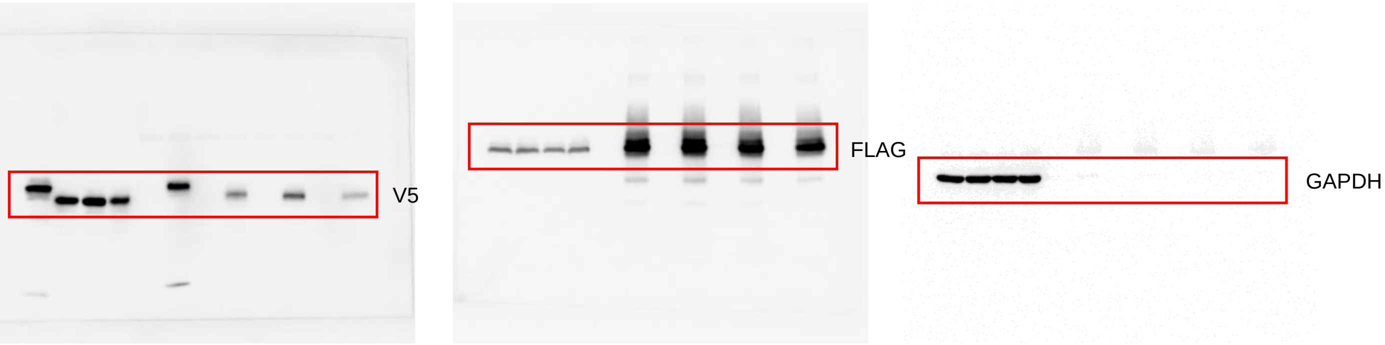

Figure 4

A

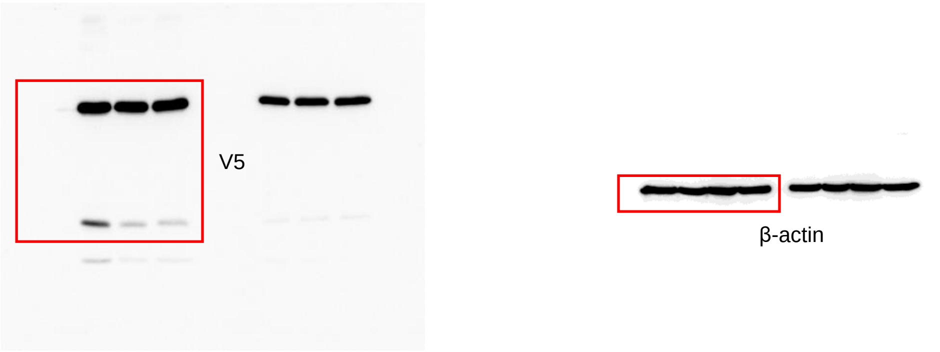

B

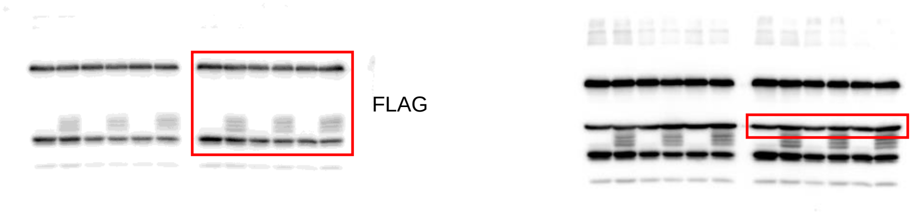

Figure 5

A

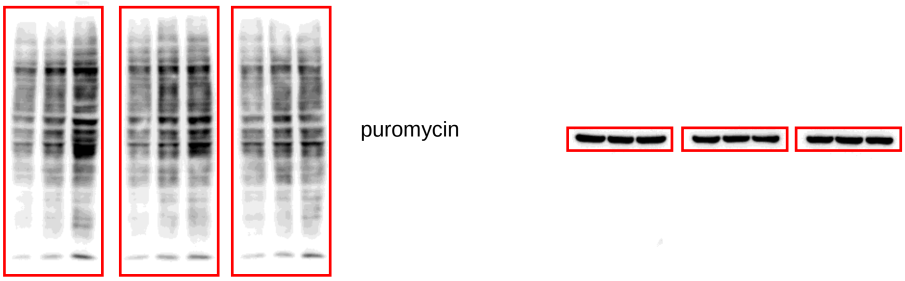

D

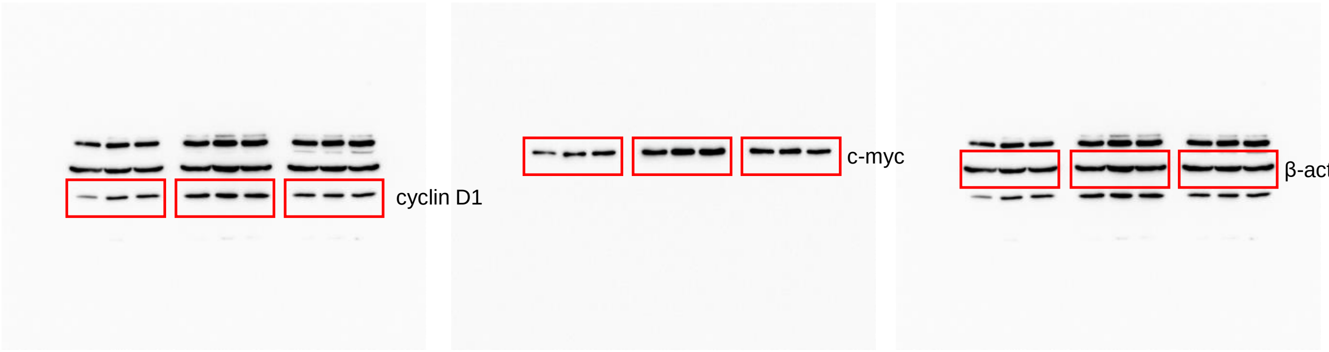

Supplementary Figure 1

B

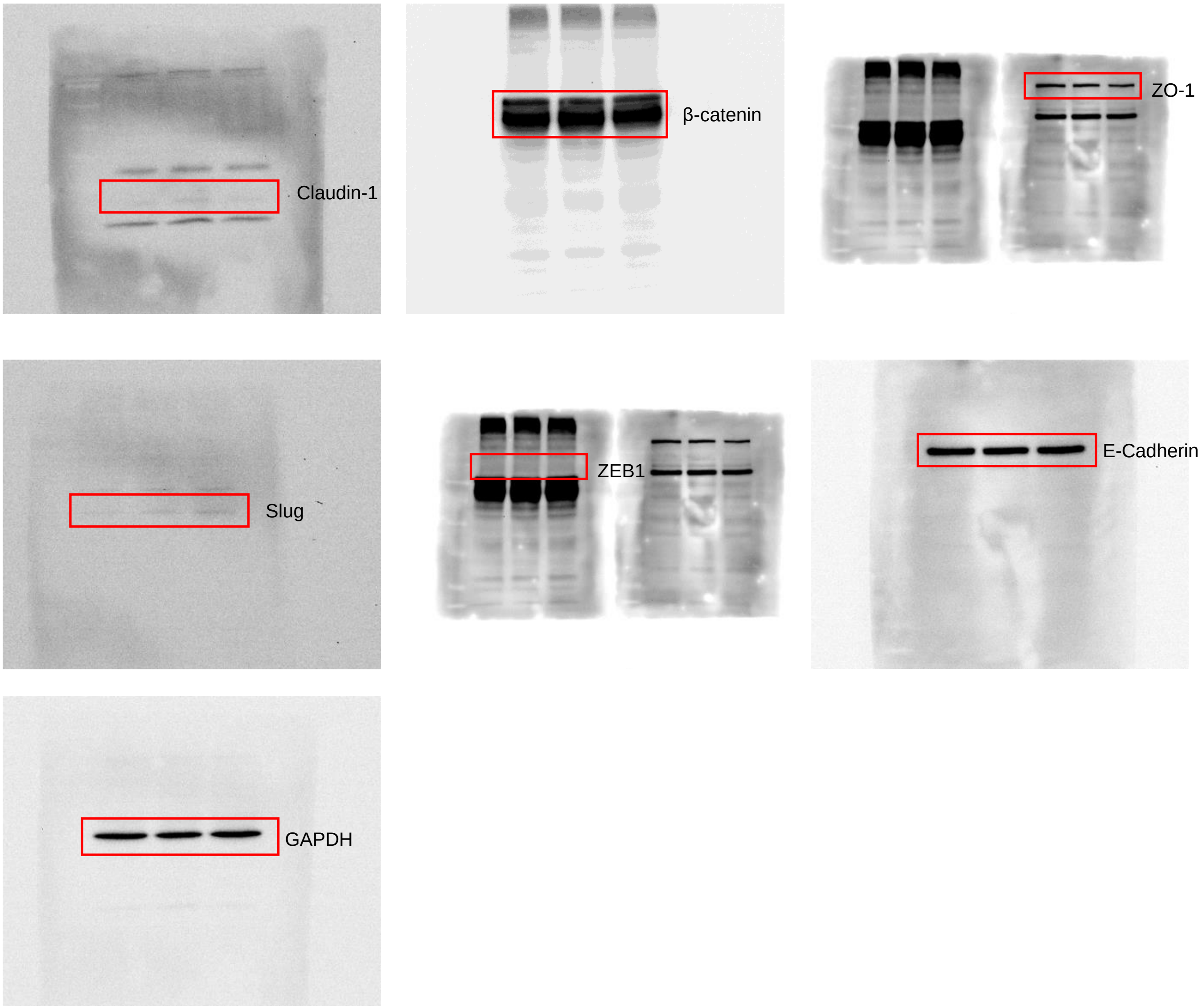

Supplementary Figure 3

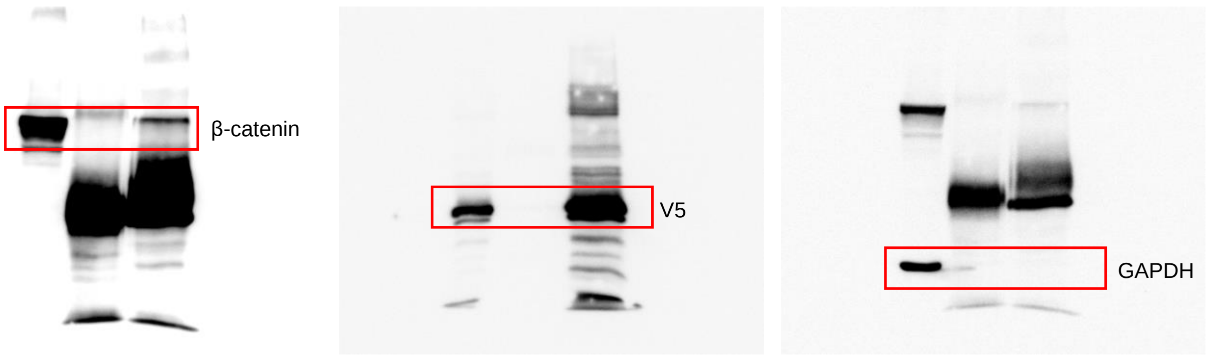

Supplement: Supplementary file 1 — Original WB file [file 41419_2025_7695_MOESM1_ESM.pdf]
