# Supplementary material for: NAG-1/GDF15 as a tumor suppressor in colorectal cancer: inhibition of β-catenin and NF-κB pathways via interaction with EpCAM
Source: Cell Death Dis. 2025 May 2;16(1):355. doi: 10.1038/s41419-025-07695-w (PMC12048721; doi:10.1038/s41419-025-07695-w)
Supplement: Supplementary file 2 — Supplementary figure (1,2,3, 4) and Tables (1, 2, 3–1, 3–2, 3–3, 4, 5) [file 41419_2025_7695_MOESM2_ESM.docx]

**Supplementary Information**

**NAG-1/GDF15 as a Tumor Suppressor in Colorectal Cancer: Inhibition of β-Catenin and NF-κB Pathways via Interaction with EpCAM**

**^1^Jaehak Lee**, **^1^Ilju Kim, ^2^Junsun Ryu, ^3^Thomas Eling, and ^1^Seung Joon Baek***.

**Supplementary Figures**


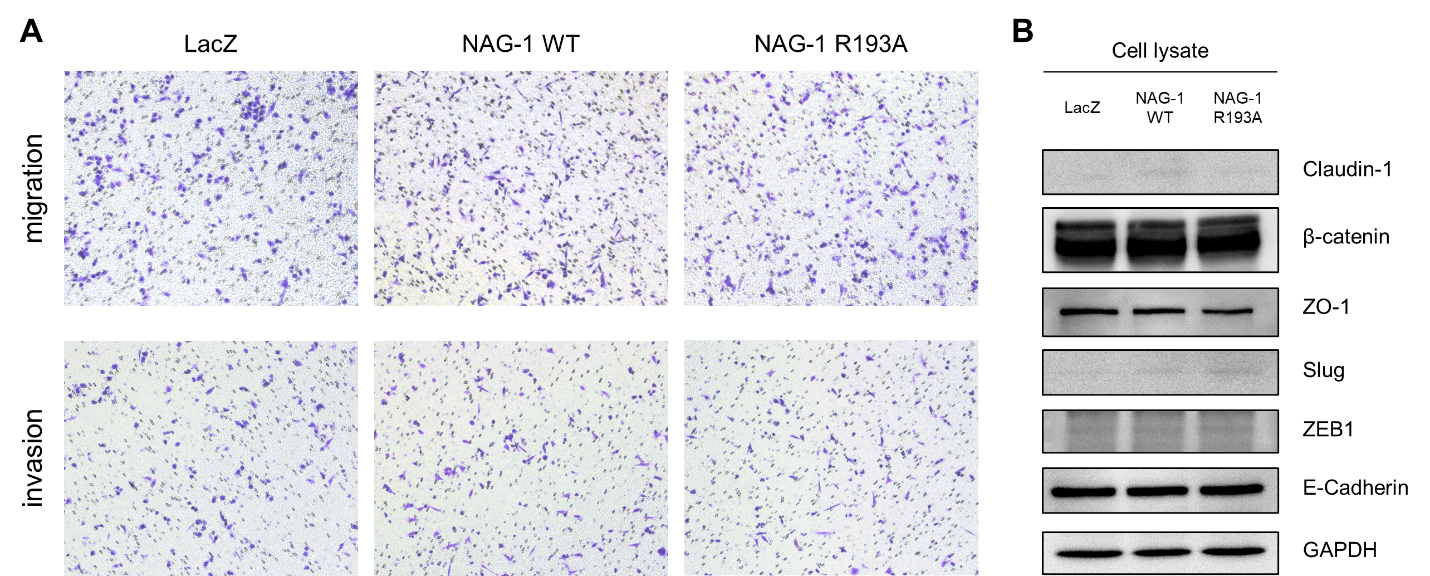


Supplementary Figure 1. NAG-1/GDF15 does not increase migration/invasion activity in colorectal cancer cells. (A) Transwell migration/invasion assay. No difference was observed between SW480 stable cell lines. (B) EMT-related protein expression in stable cell lines. NAG-1 WT and R913A did not increase protein level regarding EMT.


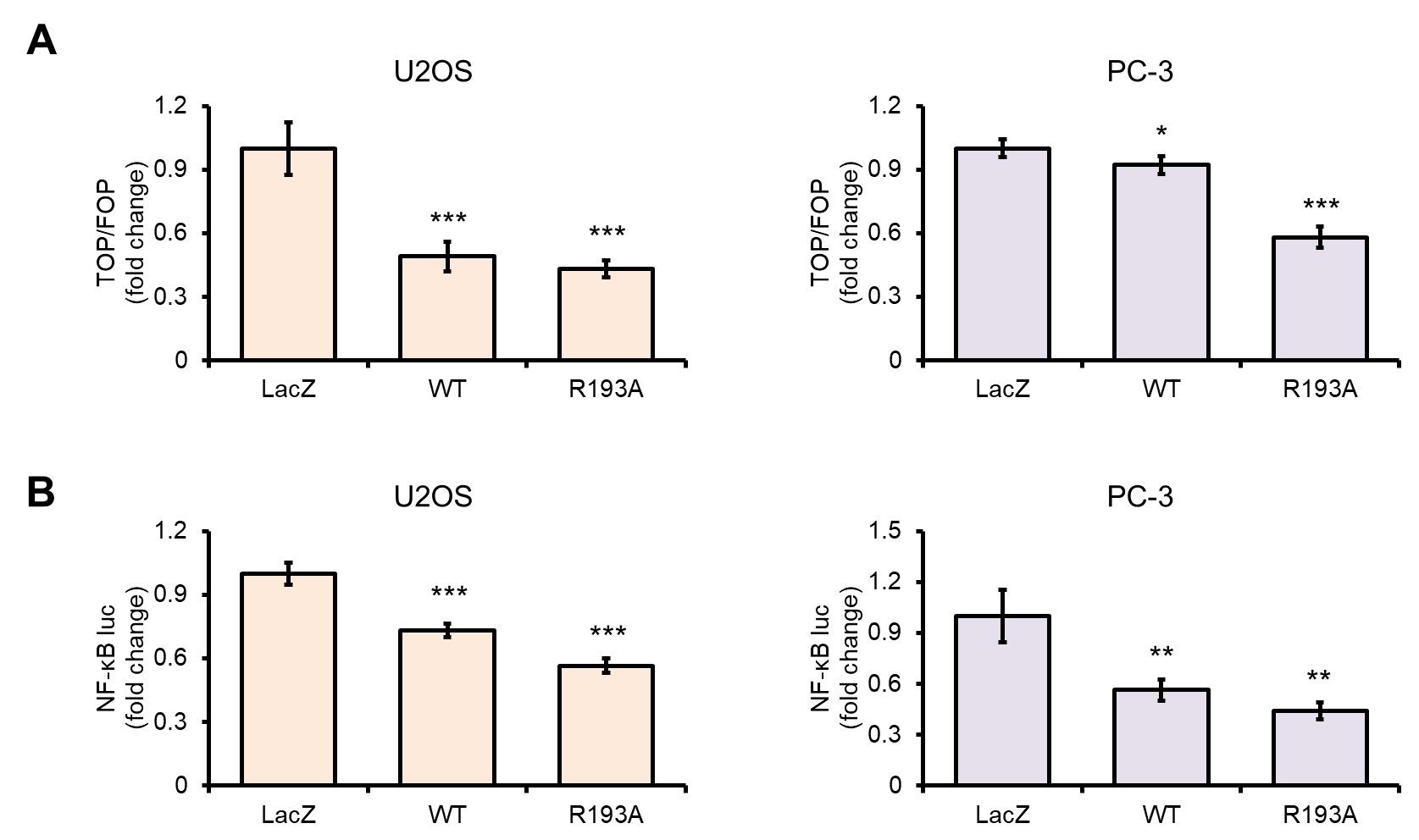


Supplementary Figure 2. NAG-1/GDF15 decreases β-catenin and NF-κB activity in osteosarcoma and prostate cancer cell lines. (A, B) Osteosarcoma cell line U2OS and prostate cancer cell line PC-3 were transfected with NAG-1/GDF15 WT or R193A plasmids, along with specific luciferase plasmids, for 24 h, followed by luciferase measurement. LacZ-transfected cells were used as a control. Graphs represent means ± SD (n=4). **p* value < 0.05, ***p* value < 0.01, ****p* value < 0.001.

Supplementary Figure 3. TCGA data regarding rectal cancer and NAG-1/GDF15. There was tendency that high NAG-1 expressing patients may have better survival than low NAG-1/GDF15 patients, although it did not show statistical significance.


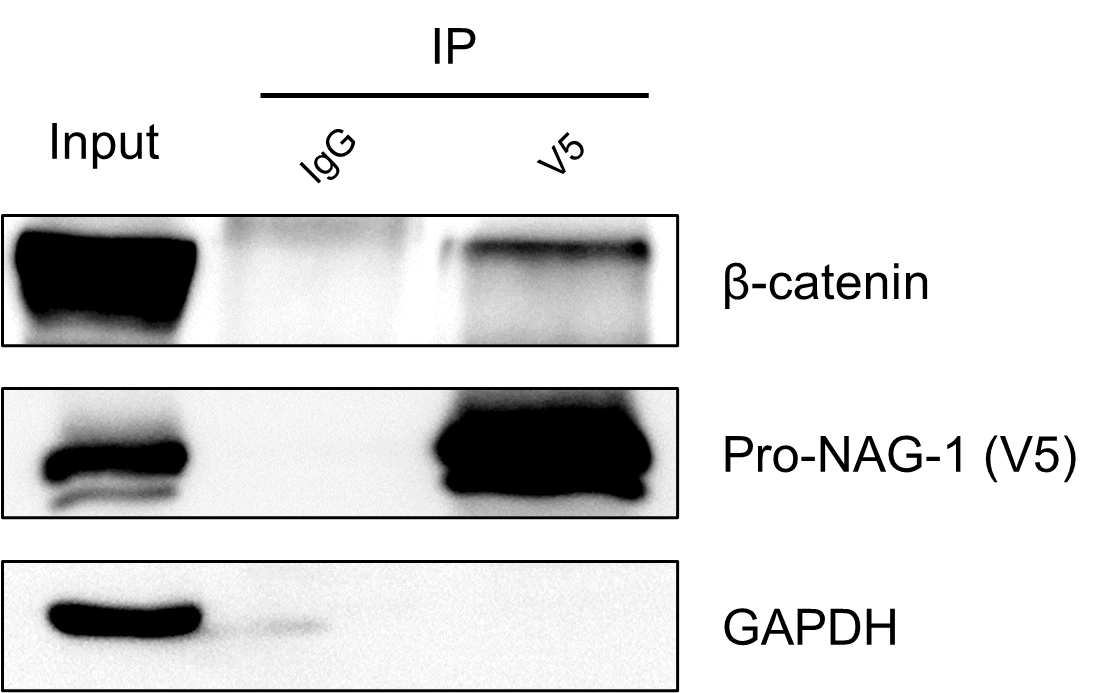


Supplementary Figure 4. NAG-1/GDF15 binds to β-catenin. NAG-1/GDF15-V5-His was transfected to HCT116 and pulled down with anti-V5 antibody. Interaction between NAG-1/GDF15 and β-catenin was observed.

**Supplementary Tables**

Supplementary table 1. Phage display screening

| Gene symbol | Gene ID | Gene full name | Protein sequence |
| --- | --- | --- | --- |
| ACSM3 | 6296 | acyl-CoA synthetase medium chain family member 3 | 523-586 |
| LTBP1 | 4052 | latent transforming growth factor beta binding protein 1 | 1022-1087 |
| EPCAM | 4072 | epithelial cell adhesion molecule | 261-314 |

Supplementary table 2. Reagents.

| Reagent | Manufacturer | Catalog number |
| --- | --- | --- |
| quercetin | Sigma-Aldrich (St. Louis, MO, USA) | Q4951 |
| GM 6001 | Sigma-Aldrich | 364205 |
| CMK | Enzo Life Sciences (Farmingdale, NY, USA) | ALX-260-022 |
| PMA | InvivoGen (San Diego, CA, USA) | tlrl-pma |
| BML-284 | MCE (Monmouth Junction, NJ, USA) | HY-19987 |
| TNF-α | Peprotech (Cranbury, NJ, USA) | 300-01A |
| puromycin | Sigma-Aldrich | P8833 |
| DAPI | Sigma-Aldrich | D9542 |
| 4% paraformaldehyde | Biosesang (Yongin, Gyeonggi-do, Korea) | PC2031-050-00 |
| compound E | MCE | HY-14176 |

Supplementary table 3-1. Antibodies used for western blot analysis.

| Antibody | Manufacturer | Catalog number | Dilution |
| --- | --- | --- | --- |
| V5 | Invitrogen (Carlsbad, CA, USA) | R960-25 | 1:5,000 |
| puromycin | Abclonal (Woburn, MA, USA) | A23031 | 1:2,000 |
| Phospho-S6 Ribosomal Protein (Ser235/236) | Cell Signaling Technology (Danvers, MA, USA) | 4858 | 1:1,000 |
| c-myc | Cell Signaling Technology | 5605 | 1:1,000 |
| cyclin D1 | Cell Signaling Technology | 2922 | 1:1,000 |
| NAG-1 | in-house [1] | N/A | 1:1,000 |
| β-actin | Santa Cruz Biotechnology (Dallas, TX, USA) | sc-47778 | 1:1,000 |
| FLAG | Sigma-Aldrich | F1804 | 1:2,000 |
| EpCAM | Cell Signaling Technology | 93790 | 1:1,000 |
| Calnexin | Cell Signaling Technology | 2679 | 1:1,000 |
| HSP90 | Cell Signaling Technology | 4877 | 1:1,000 |
| GAPDH | Santa Cruz Biotechnology | sc-365062 | 1:1,000 |
| Claudin-1 | Cell Signaling Technology | 4933 | 1:1,000 |
| β-catenin | Cell Signaling Technology | 8480 | 1:1,000 |
| ZO-1 | Cell Signaling Technology | 8193 | 1:1,000 |
| Slug | Cell Signaling Technology | 9585 | 1:1,000 |
| ZEB1 | Cell Signaling Technology | 3396 | 1:1,000 |
| E-Cadherin | Cell Signaling Technology | 3195 | 1:1,000 |
| Anti-rabbit IgG, HRP-linked Antibody | Cell Signaling Technology | 7074 | 1:5,000 |
| Goat anti-Mouse IgG (H+L) Secondary Antibody, HRP | Invitrogen | 62-6520 | 1:5,000 |

Supplementary table 3-2. Antibodies used for co-immunoprecipitation.

| Antibody | Manufacturer | Catalog number | Used per reaction |
| --- | --- | --- | --- |
| V5 | Abclonal | AE092 | 1 μg |
| FLAG | Abclonal | AE089 | 1 μg |
| Normal Rabbit IgG | Sigma-Aldrich | 12-370 | 1 μg |

Supplementary table 3-3. Antibodies used for immunocytochemistry and immunohistochemistry.

| Antibody | Manufacturer | Catalog number | Dilution |
| --- | --- | --- | --- |
| FLAG | Sigma-Aldrich | F1804 | 1 μg/ml |
| PCNA | Santa Cruz Biotechnology | sc-56 | 1:50 |
| Goat anti-Mouse IgG (H+L) Cross-Adsorbed Secondary Antibody, Alexa Fluor™ 647 | Invitrogen | A-21235 | 1 μg/ml |

Supplementary table 4. Primers used for qRT-PCR.

| Primer | Sequence (5’ 🡪 3’) |
| --- | --- |
| 18S rRNA F | CGG CGA CGA CCC ATT CGA AC |
| 18S rRNA R | GAA TCG AAC CCT GAT TCC CCG TC |
| c-myc F | CCT GGT GCT CCA TGA GGA GAC |
| c-myc R | CAG ACT CTG ACC TTT TGC CAG G |
| cyclin D1 F | TCT ACA CCG ACA ACT CCA TCC G |
| cyclin D1 R | TCT GGC ATT TTG GAG AGG AAG TG |
| VEGF-A F | TTG CCT TGC TGC TCT ACC TCC A |
| VEGF-A R | GAT GGC AGT AGC TGC GCT GAT A |
| NOS2 F | GCT CTA CAC CTC CAA TGT GAC C |
| NOS2 R | CTG CCG AGA TTT GAG CCT CAT G |
| PPAR δ F | GGC TTC CAC TAC GGT GTT CAT G |
| PPAR δ R | CTG GCA CTT GTT GCG GTT CTT C |
| MMP7 F | TCG GAG GAG ATG CTC ACT TCG A |
| MMP7 R | GGA TCA GAG GAA TGT CCC ATA CC |
| ICAM-1 F | AGC GGC TGA CGT GTG CAG TAA T |
| ICAM-1 R | TCT GAG ACC TCT GGC TTC GTC A |
| uPA F | GGC TTA ACT CCA ACA CGC AAG G |
| uPA R | CCT CCT TGG AAC GGA TCT TCA G |
| MCP-1 F | AGA ATC ACC AGC AGC AAG TGT CC |
| MCP-1 R | TCC TGA ACC CAC TTC TGC TTG G |
| IL-1β F | CCA CAG ACC TTC CAG GAG AAT G |
| IL-1β R | GTG CAG TTC AGT GAT CGT ACA GG |
| IL-6 F | AGA CAG CCA CTC ACC TCT TCA G |
| IL-6 R | TTC TGC CAG TGC CTC TTT GCT G |
| IL-8 F | GAG AGT GAT TGA GAG TGG ACC AC |
| IL-8 R | CAC AAC CCT CTG CAC CCA GTT T |

Supplementary table 5. Primers used for cloning and site-directed PCR mutagenesis.

| Primer | Sequence (5’ 🡪 3’) |
| --- | --- |
| EpCAM HindIII F | TAA TTA AAG CTT ATG GCG CCC CCG CAG GTC CTC GCG T |
| EpCAM XhoI R | TGC GGC CTC GAG TGC ATT GAG TTC CCT ATG CAT CTC A |
| EpCAM ΔEpICD F | CTC GAG TAC GAC TAC AAA G |
| EpCAM ΔEpICD R | AAT AAC CAG CAC AAC AAT TC |
| EpICD ΔEpCAM F | TCC AGA AAG AAG AGA ATG G |
| EpICD ΔEpCAM F | CAT AAG CTT GGC AGC |
| EGFP XhoI F | AAT TAA CTC GAG GTG AGC AAG GGC GAG GAG CT |
| EGFP XhoI R | CGT TGA CTC GAG CTT GTA CAG CTC GTC CAT GC |
| NAG-1 Δ197-231 F | CCA CGG GAG GTG CAA G |
| NAG-1 Δ197-231 R | ACG CGC TCT GCG GC |
| NAG-1 Δ232-268 F | GTG CCA GCG CCC TG |
| NAG-1 Δ232-268 R | CGA CAG CAC CCA ATC G |
| NAG-1 Δ269-308 F | AGT CTA GAG GGC CC |
| NAG-1 Δ269-308 R | CGT GTC GGG CTT CAG |

1. Baek SJ, Kim K-S, Nixon JB, Wilson LC, Eling TE. Cyclooxygenase Inhibitors Regulate the Expression of a TGF-β Superfamily Member That Has Proapoptotic and Antitumorigenic Activities. *Mol Pharmacol* 2001, **59**(4)**:** 901-908.
